# Network Approaches for Dissecting the Immune System

**DOI:** 10.1016/j.isci.2020.101354

**Published:** 2020-07-10

**Authors:** Hao Shi, Koon-Kiu Yan, Liang Ding, Chenxi Qian, Hongbo Chi, Jiyang Yu

**Affiliations:** 1 Departments of Computational Biology, St. Jude Children's Research Hospital, Memphis, TN 38105, USA; 2 Department of Immunology, St. Jude Children's Research Hospital, Memphis, TN 38105, USA

**Keywords:** Biological Sciences, Immunology, Systems Biology

## Abstract

The immune system is a complex biological network composed of hierarchically organized genes, proteins, and cellular components that combat external pathogens and monitor the onset of internal disease. To meet and ultimately defeat these challenges, the immune system orchestrates an exquisitely complex interplay of numerous cells, often with highly specialized functions, in a tissue-specific manner. One of the major methodologies of systems immunology is to measure quantitatively the components and interaction levels in the immunologic networks to construct a computational network and predict the response of the components to perturbations. The recent advances in high-throughput sequencing techniques have provided us with a powerful approach to dissecting the complexity of the immune system. Here we summarize the latest progress in integrating omics data and network approaches to construct networks and to infer the underlying signaling and transcriptional landscape, as well as cell-cell communication, in the immune system, with a focus on hematopoiesis, adaptive immunity, and tumor immunology. Understanding the network regulation of immune cells has provided new insights into immune homeostasis and disease, with important therapeutic implications for inflammation, cancer, and other immune-mediated disorders.

## Graphical Abstract

**Figure:**
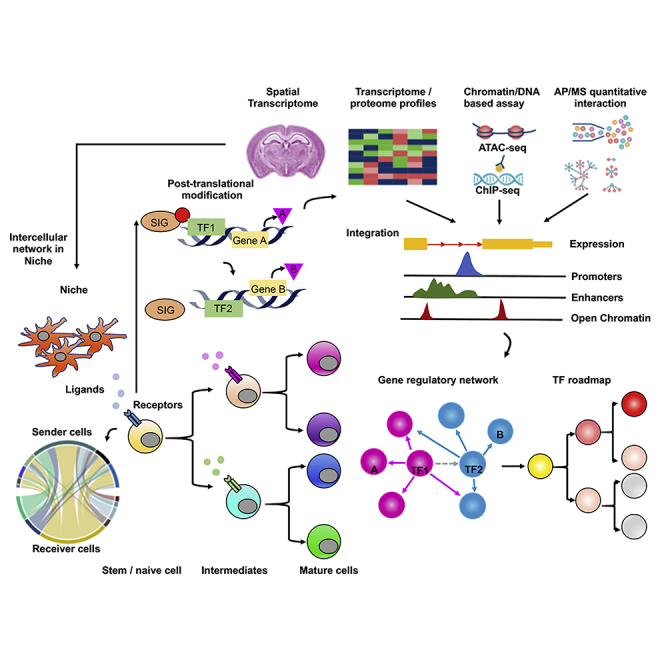

## Introduction

The mammalian immune system maintains a balance between inducing an appropriate immune response to exogenous pathogens and avoiding autoimmune reactions to self-antigens. This balance is achieved by a close collaboration of innate and adaptive immune cells, which could undergo changes in functional status in response to inflammatory conditions or a tissue-specific milieu. For instance, in adaptive immunity, T and B lymphocytes are the two main players responsible for cell-mediated immune response and antibody response, respectively. CD4^+^-naive T cells can differentiate into T helper cells upon antigen stimulation (Saravia et al., 2019), and regulatory T (Treg) cells can gain tissue-specific signatures to become specific types of tissue-resident Treg cells (Shi and Chi, 2019), both of which require antigen presentation and cytokine stimulation provided by innate immune cells like dendritic cells (DCs) (Eisenbarth, 2019). Remarkably, these innate and adaptive immune cells are produced through a series of developmental steps called hematopoiesis, which is sustained throughout life by a relatively small pool of hematopoietic stem cells (HSCs) that reside in the marrow. These immune cells differentiated from HSCs to specialized cell types and work in concert to maintain immune homeostasis. However, the heterogeneous cellular phenotypes and functions of these immune cells in different tissues or tumors are still not fully explored. Moreover, the immune cell differentiation requires the orchestration of complex signaling networks and transcriptional regulation at the intracellular level. Other types of cells in close proximity are also critical for instructing the differentiation. Characterizing these multilayer cellular networks is vital to manipulate the immune system to harness its unique therapeutic potential, and how to achieve this characterization remains an important question in the field.

The recent advances in next-generation sequencing (NGS)-based omic technologies, such as bulk RNA sequencing (RNA-seq), assay for transposase-accessible chromatin using sequencing (ATAC-seq) (Buenrostro et al., 2015), and chromatin immunoprecipitation sequencing (ChIP-seq) (Park, 2009) have provided us powerful tools to dissect the transcriptome at the transcript level, chromatin accessibility, and transcription factor (TF) binding regions on the chromatin. These efforts focused on understanding gene regulatory network at the population level; however, they failed to capture the heterogeneous nature of cells, which is particularly the case for the immune system highlighted by its vast number of constituents and their functional states. The development of single-cell technologies over the past few years, including single-cell RNA-seq (scRNA-seq) and single-cell ATAC-seq (scATAC-seq) (Mezger et al., 2018) have transformed our understanding of transcriptome and chromatin accessibility in a higher resolution (Giladi and Amit, 2018; Stubbington et al., 2017). Meanwhile, newly developed spatial transcriptomics further provides the spatial location information for each cell type in the tissue (Moncada et al., 2020; Stahl et al., 2016), while coupling with protein-level measurement at the same time such as cellular indexing of transcriptomes and epitopes by sequencing (CITE-seq) further complements the detection of single-cell transcriptome to minimize the effect from discrepancy between gene and protein level (Stoeckius et al., 2017). Together with the development of a variety of computational methodologies, we have started to have a glimpse of the system-wide cellular networks, such as intracellular transcriptional regulatory and signaling networks, and extracellular communication networks. Here we summarize the latest progress in omics technologies and computational methodologies that facilitates the reconstruction and inference of different kinds of molecular networks. We will start with the population-level approaches and then highlight the latest development in the single-cell approaches. It is worthwhile to mention that all different kinds of molecular networks, despite the contextual differences, could be abstracted into nodes and edges, in which the nodes are molecules, such as genes, proteins, or cells, and the edges can depict connections between different entities. The highly structured source-target relationships facilitate data integration and permit effective visualization of the underlying patterns. In this review, we emphasize the importance of “thinking networks” by highlighting several applications in immunology, especially regarding hematopoiesis, adaptive immunity, and tumor immunology.

## Omics Technologies and Computational Methodologies for Network Inference

**Figure 1:**
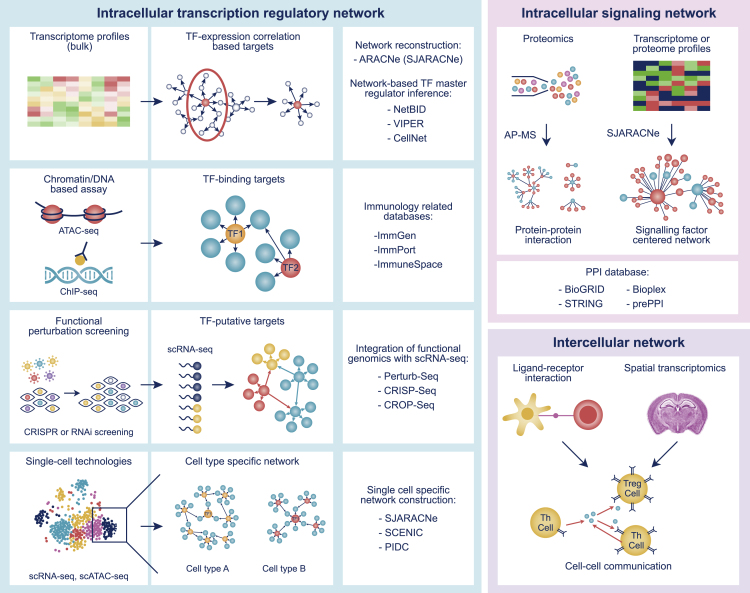
An Overview of Common Omics Technologies and Computational Methodologies for Network Inference This figure shows three major types of molecular network, including intracellular transcriptional regulatory networks (left panel), intracellular signaling networks (upper right panel), and intercellular (cell-cell) communication networks (lower right panel), as well as how they can be inferred from various omics technologies. The left panel shows four different approaches to transcriptional regulatory network inference: (1) constructing a gene co-expression network from transcriptome profiles; (2) curating a transcription factor network from binding targets identified by chromatin or DNA-based assays such as ATAC-seq and ChIP-seq; (3) identifying putative targets from genome-wide perturbation methods followed by single-cell RNA sequencing to construct a gene regulatory network; and (4) using scRNA-seq to construct cell-type-specific gene regulatory networks. The upper right panel illustrates two approaches to signaling network inference: one uses direct proteomics profiling through AP-MS to construct protein-protein interaction networks, whereas the other uses computational methods such as SJARACNe to infer a signaling factor-centered network from proteomics data. In the lower right panel, a simple diagram shows common strategies for intercellular network construction. Cell-cell interactions are estimated from the expression levels of ligand-receptor pairs, with location information (obtained by spatial transcriptomics) also taken into account.

Recent technological advances in high-throughput omics technologies offer unprecedented opportunities to probe the complex interactions between DNA or mRNAs and TFs or signaling proteins. There has been a rapid growth of computational methodologies developed to analyze and exploit the large amount of multimodal genomic data being generated, resulting in the reconstruction and inference of various molecular networks, operating at various molecular levels, space, and timescales. In this section, we summarize the key technologies and methodologies that are particularly important for understanding the underlying networks (Figure 1).

### Integration of Various Genomic Data to Reverse Engineer Transcriptional Regulatory Networks

#### Transcriptome Profiling

Transcriptome profiling by microarray or, nowadays, by NGS (RNA-seq) is one of the most prevalent technologies used to understand cellular systems; therefore, it has been widely used to provide insights into hematopoiesis and immunology. Table 1 summarizes the use of large-scale gene expression profiling in hematopoiesis-related studies. By measuring the system-wide level of gene expression at different time points or under different conditions, transcription profiling serves as the basis for inferring the relations among genes and TFs, i.e., the transcriptional regulatory networks. There are essentially two kinds of regulatory relations (edges). One is co-expression, as analyzed by methods such as weighted gene correlation network analysis (WGCNA) (Langfelder and Horvath, 2008), based on Pearson or Spearman correlations. However, many co-expression relations are rather indirect or redundant. To overcome this problem, algorithm for the reconstruction of accurate cellular networks (ARACNe) (Margolin et al., 2006) and a recent implementation thereof named SJARACNe (Khatamian et al., 2019) use mutual information to capture nonlinear gene-gene relations and apply data-processing inequality to remove redundant edges. Nevertheless, in practice, the most important application of a network is not singling out a particular edge but identifying regulons or regulatory programs. A regulon is a set of genes that is regulated by a TF and is presumed to be responsible for a common biological function. The TF is referred to as a driver or master regulator. In many cases, the drivers are referred to as hidden drivers because although they are responsible for driving the transcriptional response to shifts between wild-type and other conditions, their expression may not differ much under the different conditions. Different algorithms have been developed to identify such drivers and their functional targets. For instance, CellNet (Morris et al., 2014) uses co-expression information, whereas both VIPER (virtual inference of protein-activity by enriched regulon analysis) (Alvarez et al., 2016) and NetBID (data-driven network-based Bayesian inference of drivers) (Du et al., 2018) use ARACNe/SJARACNe-derived regulatory networks to infer protein activities in individual samples and master regulators associated with phenotypes.

**Table 1:** Recent System-wide Gene Expression Datasets for Inferring Transcription Regulatory Networks of Hematopoiesis

		Description	Reference	Accession
Gene expression	*Mus musculus*	Immunological Genome Project gene expression profiling for 103 different immunocytes by RNA-seq. Immune cell populations were isolated with high purity by flow cytometry.	Yoshida et al. (2019)	GSE109125
		Total RNA was obtained from 54 hematopoietic cells types and eight non-hematopoietic out-groups for microarray.	de Graaf et al., 2016	GSE77098
		Comparison of gene expression in different hematopoietic cell types (RNA-seq).	Choi et al., 2019	GSE116177
		Transcriptional heterogeneity and lineage commitment in myeloid progenitors (scRNA-seq).	Paul et al. (2015)	GSE72857
		Transcriptional plasticity, priming, and commitment in hematopoietic lineages. Bone marrow hematopoietic progenitor mRNA profiles from single cells were generated by deep sequencing of thousands of single cells (scRNA-seq).	Giladi et al. (2018)	GSE92575
		The transcriptional landscape of mouse blood stem/progenitor cell transitions at single-cell resolution (scRNA-seq).	Nestorowa et al., 2016	GSE81682
		Gene expression in hematopoietic stem and progenitor cells from Gata1-EGFP reporter mouse bone marrow (microarray).	Drissen et al., 2016	GSE49241
		Distinct myeloid progenitor differentiation pathways uncovered through scRNA-seq.		GSE77029
		Identification of subset-specific dendritic cell progenitors revealing early commitment in the bone marrow (scRNA-seq).	Schlitzer et al., 2015	GSE60781
		scRNA-seq profiling of common myeloid progenitor (CMP) cells.	Olsson et al., 2016	GSE70236
		scRNA-seq profiling of hematopoietic progenitors from mouse bone marrow and fetal liver.	Tusi et al., 2018	GSE89754
	*Homo sapiens*	Distinct routes of lineage development reshape the human blood hierarchy across ontogeny. Using SMART-seq, the expression profile of the hematopoietic stem cells and other hematopoietic progenitors (12 samples in duplicates) were analyzed.	Notta et al. (2016)	GSE76234
		scRNA-seq of human hematopoietic stem and progenitors (HSPCs). Lin^−^CD34^+^CD38^+^ progenitors and Lin^−^CD34^+^CD38^−^ stem cell-enriched HSPCs were individually sorted, their surface marker fluorescence intensities were recorded, and they were subjected to scRNA-seq.	Velten et al. (2017)	GSE75478
		Comparison of gene expression in different human hematopoietic cell types (bulk RNA-seq).	Choi et al., 2019	GSE115736
		The expression profiles of fetal and adult human erythroblasts at two differentiation stages by bulk RNA-seq.	Huang et al., 2017	GSE102182
		mRNA profiles of fetal and adult erythroblasts were generated by bulk RNA-seq.	Lalonde et al., 2017	GSE90878
		Transcriptional profiling in human primary fetal and adult CD34^+^ HSPCs and erythroid progenitor cells by RNA-seq.	Liu et al., 2017	GSE74053
		Single-cell epigenomics and transcriptomics mapping the continuous regulatory landscape of 10 cell types of human hematopoietic differentiation (scRNA-seq).	Buenrostro et al. (2018)	GSE96811
		Transcription profiles of hematopoietic and leukemic cell types assayed using unstranded RNA-seq, across 13 normal hematopoietic cell types and three acute myeloid leukemia cell types. The complete dataset contains a total of 81 samples.	Corces et al. (2016)	GSE74246
		CD34^+^-derived erythroblasts grown in *in vitro* culture were sorted using surface markers and processed using bulk RNA-seq.	Ludwig et al., 2019	GSE115678
		Developmental differences between neonatal and adult human erythropoiesis (bulk RNA-seq).	Yan et al., 2018	GSE107218
		RNA-seq profiles of eight primary human hematopoietic progenitor populations representing the major myeloid commitment stages and the main lymphoid stages.	Chen et al., 2014	EGAD00001000745

Systemic transcriptome profiling of mouse and human hematopoietic populations using microarray, bulk and single-cell (sc) RNA-seq was collected from literatures and sorted into the table. The accession numbers initiated with GSE are from NCBI Gene Expression Omnibus (GEO), whereas the one initiated with EGA is from European Genome-phenome Archive (EGA). The datasets were found by searching the keyword “hematopoiesis” in NCBI GEO and EGA datasets portal uploaded in the previous 6 years, filtered by the RNA sample type and manually refined.

#### Integration of Chromatin/DNA-Based Assays Such as ATAC-Seq and ChIP-Seq

The inference of transcriptional regulatory networks can be greatly facilitated by DNA-based NGS assays. ChIP-seq is widely used to study the binding sites of TFs at the genome-wide level (Furey, 2012), and it has been eagerly adopted in immunology (Northrup and Zhao, 2011). Consortium-wide efforts have sought to construct “wiring diagrams” by combining the binding events of many TFs (Gerstein et al., 2012). Nevertheless, performing ChIP-seq experiments on more than a thousand TFs is not very practical; more feasible approaches use assays for profiling open chromatin, such as DNAse1 hypersensitivity assays (Vierstra and Stamatoyannopoulos, 2016) and ATAC-seq (Buenrostro et al., 2013), together with TF-binding motifs (Neph et al., 2012; Rendeiro et al., 2016). Large-scale consortia efforts such as ENCODE and RoadMap (Roadmap Epigenomics Consortium et al., 2015) have generated a vast amount of functional genomic data for this purpose, including data from samples specifically related to hematopoiesis such as CD34^+^ cells. Several databases focused on immunology applications can be found in the literature; they include ImmGen (Shay and Kang, 2013), ImmPort (Bhattacharya et al., 2014), and ImmuneSpace (Sauteraud et al., 2016). There have been numerous efforts to develop algorithms for integrating chromatin-based assays and gene expression data (Chaudhri et al., 2020; Duren et al., 2017; Miraldi et al., 2019; Ramirez et al., 2017; Yoshida et al., 2019), and some groups have integrated data from chromosomal interactions assays (Mifsud et al., 2015; Mumbach et al., 2016), taking into account enhancer-promoter contacts (Schoenfelder and Fraser, 2019).

#### Pooled Functional RNAi or CRISPR Perturbation Screening

A direct way to identify the regulatory targets of a TF is to perform a knockout experiment and then examine the genes with highly differential expression. RNAi, a high-throughput functional perturbation screening technique exploiting gene silencing mechanisms, has been widely used for a decade (Boutros and Ahringer, 2008), and computational approaches such as Bayesian networks have also been studied (Tegnér and Björkegren, 2007). Nevertheless, the differential expression of genes might be the result of indirect regulation and, therefore, not always consistent with the direct binding targets identified in ChIP-seq or ATAC-seq experiments. More recently, the RNA-guided CRISPR-associated Cas9 nuclease has been combined with genome-scale guide RNA libraries for unbiased, phenotypic screening based on cell lethality or growth (Shalem et al., 2015), and this approach has been applied to studying cancer therapy (Wei et al., 2019). CRISPR and conventional RNAi screens have been shown to perform comparably in identifying essential genes (Morgens et al., 2016). Novel CRISPR interference/activation technologies provide a complementary approach to RNAi by repressing or activating gene expression at the transcriptional level, whereas RNAi represses gene expression at the mRNA level (Gilbert et al., 2014). A single perturbation of a TF by RNAi or CRISPR followed by bulk RNA-seq profiling of cells with or without the perturbation is commonly used to identify the putative targets of the TF, thus defining its transcriptional regulatory network. However, it is extremely resource consuming to scale this approach up to the genome-wide level. Recently, technologies that combine a pooled CRISPR screen with scRNA-seq, such as Perturb-seq (Dixit et al., 2016), CRISP-seq (Jaitin et al., 2016), and CROP-seq (Datlinger et al., 2017), have been introduced. By using scRNA-seq as the readout, the genome-wide transcriptional regulatory network can be reconstructed.

#### Single-Cell Technologies Profile Cell-type-specific Multimodal Measurements

In the last few years, advances in the technologies of cell suspension, automation, and microfluidics and the implementation of unique molecular identifiers have pushed single-cell genomics to an unprecedented level (Tanay and Regev, 2017). scRNA-seq is the most widely used single-cell assay (Luecken and Theis, 2019; Stegle et al., 2015), and considerable effort has been invested in profiling the entire human and mouse cell atlases (Adlung and Amit, 2018; Han et al., 2018; Regev et al., 2017). Method development is an exciting area, and algorithms focused on clustering (Kiselev et al., 2019) and trajectory inference (Saelens et al., 2019) have been developed, applied, and reviewed. For network inference, the principles of inferring transcriptional regulatory networks from scRNA-seq data are the same as those for inference from bulk RNA-seq data. A few general methods originally developed for bulk data, such as ARACNe/SJARACNe, Bayesian networks, and partial correlation, have been applied to scRNA-seq data, and their performance has recently been reviewed (Chen and Mar, 2018). Single-cell data present unique challenges, such as the dropout effect, and a couple of algorithms have recently been developed to address this. For instance, SCENIC uses databases of *cis*-regulatory sequences to identify regulons (Aibar et al., 2017). Working at the regulon level is better than working at the single-gene level because of the reduced dimensionality. SCENIC demonstrated that the inferred network improves clustering and reduces batch effects. Other methods include PIDC, based on multivariate information theory (Chan et al., 2017), and SCODE, which uses time-series data for dynamic inference (Matsumoto et al., 2017). Of particular interest when using scRNA-seq data for network inference is the possibility of reconstructing cell-type-specific regulatory networks, because cells of a specific type can be identified first before applying the algorithm for network inference.

Like bulk RNA-seq data, scRNA-seq can be further integrated with DNA-based assays to improve network reconstruction. scATAC-seq has already been widely used to probe chromatin accessibility at the single-cell level (Clark et al., 2016; Cusanovich et al., 2018; Mezger et al., 2018), and the accessibility information can be used to identify cellular states (Bravo González-Blas et al., 2019). Further computational efforts are required to match the clusters obtained from scATAC-seq and scRNA-seq data for network inference. Nevertheless, from a technological development standpoint, more sophisticated protocols have been developed to measure accessibility and gene expression simultaneously in single cells (Buenrostro et al., 2018; Liu et al., 2019a, 2019b). Like their bulk counterparts, the burgeoning technologies of single-cell ChIP-seq and single-cell Hi-C (all-vs-all chromosome conformation capture by sequencing) will be used in the future to refine regulatory networks with single-cell resolution (Rotem et al., 2015; Stevens et al., 2017). We expect future efforts to focus on integrating single-cell omics data across different modalities (Efremova and Teichmann, 2020; Jansen et al., 2019).

### Integration of Various Genomic Data to Reconstruct Signaling Networks

Like all biological processes, hematopoiesis and the immune system are regulated on different location and timescales via different molecular networks. Transcriptional regulatory networks operate on a longer timescale, as the cellular response via transcription takes time to develop. Therefore, it is also instructive to infer and reconstruct signaling networks that regulate the cellular response on a much faster timescale. By specifying the required set of signaling factors, the SJARACNe algorithm can be used to infer signaling networks based on gene expression data. Nevertheless, the expression levels of mRNA and protein can differ substantially for many genes, especially during the temporal delay between transcription and translation (Liu et al., 2016); therefore, it is more accurate to measure the protein abundance directly. With the recent advances in mass spectrometry (MS) analytical technologies (Aebersold and Mann, 2016; Mann et al., 2013), in-depth proteomic profiling can now identify more than 10,000 proteins (by whole proteomics) and 30,000 phosphopeptides (by phosphoproteomics) across multiple samples simultaneously (Stewart et al., 2018; Tan et al., 2017; Zeng et al., 2018; Zhang et al., 2014). The measurement of phosphopeptides provides an extra layer of information on posttranslational modifications that are crucial for regulation. Recently, similar advanced MS-based platforms have been used to profile the metabolome (Lai et al., 2018) and lipidome (Geiger et al., 2016; Yang and Han, 2016). As current MS-based proteomics technologies can provide comprehensive characterizations of proteome dynamics, algorithms such as SJARACNe can use the protein abundance to construct signaling networks. Nevertheless, technologies for mapping protein-protein interactions (PPIs) serve as a complementary approach. Proteomics by affinity purification-MS is commonly used to identify PPIs (Huttlin et al., 2017). Earlier work was performed in cell lines and required the overexpression of the so-called bait, the known components of signaling proteins used for extracting the unknown proteins. Recently developed proximity-labeling methods such as BioID capture a greater number of transient interactions between baits and preys when compared with antibody-based immunoprecipitation, thus providing unprecedented resolution of protein regulatory networks (Roux et al., 2013). To reduce false-positives, quantitative interaction proteomics were recently developed to characterize directly the protein interaction network in primary immune cells (Shi et al., 2018, 2019; Voisinne et al., 2019). Moreover, integrating the cellular abundance of the interacting proteins and their interaction stoichiometry provided a quantitative and contextual view of each interaction found, permitting anticipation of whether ablating a single interacting protein could impinge on the whole protein interaction network (Hein et al., 2015; Voisinne et al., 2019). Other methods, such as removing the CRAPome, would also reduce false-positive interactions (Mellacheruvu et al., 2013), but further experimental validations are required. Algorithmically, there are many curated databases for genome-wide PPI networks, such as BioGRID (Stark et al., 2006) and STRING (Szklarczyk et al., 2019), and computational approaches that effectively integrate various omics data to identify the direction of information flow are still under active investigation (Kholodenko et al., 2012; Saez-Rodriguez et al., 2015; Vinayagam et al., 2011).

Similar to transcriptomics, proteomics has entered the era of single-cell resolution. Mass cytometry, with implementations such as cytometry by time of flight (CyTOF) or nano-LC (liquid chromatography)-MS, is increasingly popular (Spitzer and Nolan, 2016; Zhu et al., 2018). Mass cytometry has been adopted for the identification and characterization of various immune cell types and states in the mammalian immune system, with emerging applications in the clinic (Hartmann et al., 2019). Nevertheless, at the current stage of development, the number of proteins that can be profiled simultaneously in a cell is only around 40 (Gut et al., 2018); this is because the number of surface markers and the availability of protein-specific antibodies are both limited. Despite these apparent drawbacks, when compared with genome-wide scRNA-seq data, there is no dropout effect and, therefore, the joint distribution of the abundance of multiple proteins can be better estimated for inferring signaling networks (Krishnaswamy et al., 2014).

### Intercellular Cell-Cell Communication Networks

Besides the intracellular transcriptional regulatory and signaling networks, cells respond to signals, such as ligands, from cellular neighbors. The extracellular cell-cell communication networks coordinate regenerative and developmental cues in hematopoiesis. A network perspective on individual cells, ligands, and receptor interactions between cell types and their spatial organization is critical to understanding intercellular communication and to facilitating protection against disease. Although early efforts in this area were based on bulk expression (Kirouac et al., 2010), single-cell technologies have provided a unique opportunity to tackle this challenge because the expression profiles of different cell types are measured simultaneously. Most methods developed for inferring intercellular communications are based on curated ligand-receptor pairs (Ramilowski et al., 2015). The essential idea is to identify interacting ligand-receptor pairs whose respective expression is high in a corresponding pair of cell types (Kumar et al., 2018; Sheikh et al., 2019; Smillie et al., 2019; Vento-Tormo et al., 2018; Zhou et al., 2017). More recently, the NicheNet method was proposed; this takes into account the changes in the downstream signaling network in the receiver cells (Browaeys et al., 2020). The idea is to use the estimated downstream signaling changes to predict the activity of the upstream receptors to identify the functionally influential cell-cell communication (Bonnardel et al., 2019). However, the activity predication is based on downstream signaling components collected from the literature in different contexts; therefore, some cell-type-specific receptor activity might not be predicated accurately.

Using the spatial information of cells, in addition to ligand-receptor pairs, makes the inference of cell-cell interactions more precise. Microdissection and scRNA-seq have been used to map the physical network of cellular interactions (Boisset et al., 2018). Algorithms such as novoSpaRc (Nitzan et al., 2019) have been proposed for tissue-space reconstruction based on scRNA-seq data. Similar methods based on the similarity of expression may help infer the proximity of two cells in the same tissue (Stewart et al., 2019). Recently, spatial transcriptomics has been used together with scRNA-seq for identifying niches in bone marrow (Baccin et al., 2020). There are essentially two technologies for spatial transcriptomics. In general, spatial transcriptomics methods based on scRNA-seq are perhaps more broadly used because they can profile whole transcriptomes. However, single-molecule fluorescence *in situ* hybridization (Eng et al., 2017; Moffitt et al., 2018) and MERFISH (Xia et al., 2019) provide an alternative imaging-based approach for high-throughput single-cell transcriptomics. Although the number of transcripts that can be visualized simultaneously is limited because of the overlap of fluorescence signals, the approach offers higher spatial resolution; even subcellular locations can be visualized (Xia et al., 2019). A surprising application of MERFISH is measuring the number of mRNA molecules in the cell nucleus and cytoplasm to quantify the so-called RNA velocity, the determination of which was originally proposed to be accomplished by measuring the relative abundance of nascent (unspliced) and mature (spliced) mRNA (La Manno et al., 2018).

### Network Analysis

#### Topological Properties of Networks

Inferring biological networks from high-throughput profiling reveals the complex sets of interactions or relations among different biological entities. To gain further insights into the immune system, it is of fundamental interest to look at the topological structures of these networks (Barabasi and Oltvai, 2004; Brohée et al., 2008). Perhaps the most obvious property of biological networks is the existence of hubs, i.e., highly connected nodes. The number of connections of a node is a measure of its centrality; other measures include its closeness centrality, PageRank, betweenness, etc. Nodes lying at the center of a network are more likely to be essential (Iacono et al., 2019; Yu et al., 2004, 2007).

**Figure 2:**
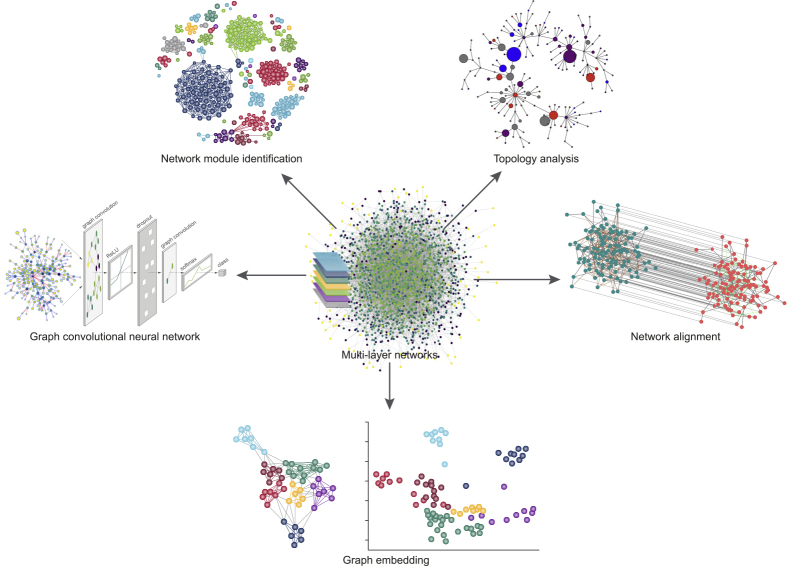
Multi-layer Network Analysis Biological systems are often represented as networks consisting of binary relations between different biological entities. With the rapid progress of omics technologies, multiple layers of networks can now be constructed. This figure summarizes the different aspects of network analysis including, but not limited to, topological analysis for identifying important nodes or edges, network module identification for finding densely connected nodes involved in the same biological functions, network alignment for uncovering highly conserved regions across multiple layers of networks, graph convolutional neural networks for node classification and link prediction, and graph embedding for projecting nodes onto a latent space.

Besides the existence of special nodes, another important property of networks is the presence of the so-called modules (Figure 2), i.e., densely connected nodes that are involved in the same biological functions (Hartwell et al., 1999). The identification of such modules has inspired various computational methods, among which the most popular (Yang et al., 2016) are random walk-based approaches, including Louvain (Subelj and Bajec, 2011), Infomap (Rosvall and Bergstrom, 2011), label propagation (Raghavan et al., 2007), and Walktrap (Pons and Latapy, 2005). Access to the various methods is generally difficult because there is no universal protocol for defining a network module, its fundamental components, or the criteria for comparing the performance of algorithms (Didier et al., 2015). The recent disease module identification DREAM challenge competition shed light on the identification and assessment of disease-relevant modules in different types of network (Choobdar et al., 2019). The network modules were accessed based on their association with complex traits and diseases by using data from genome-wide association studies. Kernel-based methods (Cao et al., 2013) and modularity quality optimization-based methods (Arenas et al., 2008; Blondel et al., 2008) performed best. An important application of network module identification is in analyzing single-cell RNA-seq and mass cytometry data (Spitzer and Nolan, 2016). In algorithms such as PhenoGraph (Levine et al., 2015) and Seurat, the high-dimensional data are transformed into nearest-neighbor graphs and the modules correspond to various cell clusters. In addition to the applications in cell population identification (Andrews and Hemberg, 2018; Cheng et al., 2019; Zhu et al., 2019), similar approaches have been used for annotating the sources of variation in single-cell data (DeTomaso et al., 2019; Luecken and Theis, 2019).

The topological analysis of networks also includes multi-network alignment (Figure 2), with the goal of finding a node mapping that uncovers highly conserved network regions (Guzzi and Milenkovic, 2018; Nelson et al., 2019). Network alignment is particularly useful in knowledge transfer between conserved regions of molecular networks across different species. Most of the popular tools, including IsoRank (Singh et al., 2008) and MAGNA (Saraph and Milenković, 2014), are designed for aligning homogeneous networks (i.e., networks containing nodes and edges of a single type). With the latest progress in omics technologies, constructing multimodal networks or heterogeneous networks, i.e., where the networks are of different types and each type may contain different biological entities, such as proteins, cells, or drugs, is more prevalent for obtaining a deeper understanding of cellular functions. Approaches to multimodal network and heterogeneous network alignment (Gu et al., 2018; Nassar and Gleich, 2017) are beginning to appear and are likely to attract more attention in the near future.

#### Machine Learning in Networks

Although it is insightful to study the topological properties of a network, a great deal of information is hidden in the interplay between network properties and various intrinsic biological properties of nodes and interactions. Machine learning is a branch of artificial intelligence in which patterns are learned from data, based on a set of mathematical rules and statistical assumptions. With the dramatically increasing speed with which large, highly complex datasets can be generated, there is growing interest in integrating machine learning and network biology to gain new perspectives and generate novel hypotheses about living systems (Camacho et al., 2018). Recent advances in omics technologies have provided numerous opportunities for systematically studying the mammalian immune system, which is composed of multiple layers of interactions. The input data to a machine learning approach consists of features extracted from multiple types of network inferred from the data, as mentioned in the preceding sections. A predictive model can be learned from the features and used to predict the various outcomes of an unseen data. A key advantage of machine learning is that it can examine multiple types or volumes of data and find patterns that would be missed by traditional approaches. Machine learning has been widely adopted for reconstructing gene regulatory networks (Mignone et al., 2019; Mochida et al., 2018) and protein-protein interaction networks (Chen et al., 2019; Franzosa et al., 2009). Recently, machine learning-based approaches have been successfully applied in the analysis of scRNA-seq data to quantify cell-cell similarity (Butler et al., 2018; Kiselev et al., 2017; Trapnell et al., 2014; Wang et al., 2017a).

As a branch of machine learning, deep learning has recently attracted considerable attention due to its advantage of learning the transformation of the data automatically rather than as defined by the researchers (Camacho et al., 2018; Kc et al., 2019; Li and Gao, 2019). However, the integration of deep learning and network biology is still at an early stage, as training a deep learning model usually requires a massive amount of data that is usually not available for a given study. Another drawback of the deep learning approach is the complex model architecture and training process that limits the interpretability of the model's predictions. Recent advances in computational approaches for network representation learning (Nelson et al., 2019) provide potential opportunities for resolving these problems (Figure 2). Instead of relying on the complicated architecture of the deep learning models, more transparent approaches have been developed to represent the networks numerically for further analysis. Early attempts at applying the graph embedding-based approaches include predicting potential interactions in various biomedical networks (Wang et al., 2017b; Zhang et al., 2018), predicting protein functions (Cho et al., 2016), and detecting differential pathways in scRNA-seq data (Costa et al., 2018).

## Immunology in a Network Perspective

Omics technologies and computational methodologies have facilitated the reconstruction and inference of various levels of molecular networks. In this section, we will highlight several areas in immunology that have greatly benefited from network approaches.

### Network Analysis to Identify Regulators Governing the Developmental Hierarchy in Hematopoiesis

**Figure 3:**
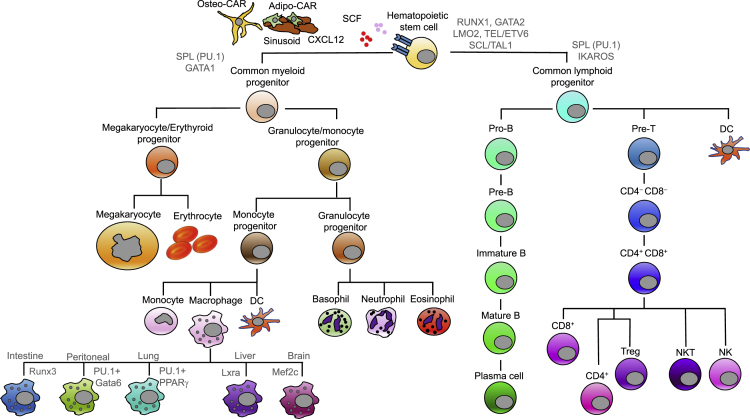
Hematopoiesis Is an Ideal Developmental Process for Network-Based Analysis In the bone marrow, hematopoietic stem cells undergo self-renewal and generate a series of progenitor cell intermediates such as common myeloid progenitors (CMPs) and common lymphoid progenitors (CLPs), which further develop into mature blood cells. CMPs give rise to megakaryocytes, erythrocytes, monocytes, macrophages, dendritic cells, or granulocytes such as basophils, neutrophils, and eosinophils. CLPs differentiate into mature B or T lymphocytes or DCs after a series of fate specifications. Notably, T cell maturation occurs in the thymus instead of in the bone marrow after some lymphoid progenitors migrate to the thymus. The whole process of hematopoiesis requires close collaboration between different cell types in the bone marrow and thymus. For instance, two kinds of mesenchymal cells, termed adipo-CAR (CXCL12-abundant-reticular) cells and osteo-CAR cells, are the main producers of the key HSC factors CXCL12 and SCF that are required to maintain the self-renewal of HSCs. The intracellular gene regulatory networks, especially the transcriptional factor (TF)-mediated gene network, are also critical for fate specification and for maintaining the equilibrium between self-renewal and differentiation. For example, TFs required for HSC formation or function and those employed in lineage-specific differentiation can be distinguished. Among the “HSC TFs” are RUNX1, GATA2, TEL/ETV6, SCL/TAL1, and LMO2. Conversely, TFs such as PU.1, GATA1, and IKAROS appear to have more important roles in lineage specification. Tissue-specific macrophage generation is also an important part of hematopoiesis in various tissues other than the bone marrow and thymus. Most tissue-specific macrophages are established prenatally from embryonic precursors. However, the tissue microenvironment regulates tissue-restricted TFs in conjunction with those ubiquitously expressed in macrophages, such as PU.1, to orchestrate tissue-specific macrophage development. Gata6 and Mef2c are, respectively, regulators of the peritoneal and microglia-specific macrophage enhancer landscapes. Other TFs, such as Lxra, Pparγ, and Runx3, are, respectively, important drivers for liver, lung, and intestinal macrophages.

As the critical players in complex immunologic networks, the different types of immune cells are produced by hematopoiesis, which is gradually established during the embryonic phase through a series of developmental steps that culminate in the generation of HSCs (Orkin and Zon, 2008). In adult organisms, the hematopoietic system is sustained by a pool of HSCs that reside in the complex microenvironment of the bone marrow (Hoggatt et al., 2016). HSCs give rise to erythroid, myeloid, and lymphoid lineages through a sequence of differentiation steps leading to mature blood cells (Figure 3). In humans, the blood-producing system produces more than 100 billion blood cells from a remarkably small pool of stem cells estimated to number 11,000 (Abkowitz et al., 2002). Thus, the equilibrium between self-renewal and differentiation in HSCs is tightly controlled by the bone marrow microenvironment and is critical for the sustained production of all blood cells. Dysregulation of this equilibrium can lead to an expansion of dysfunctional blood cells, eventually resulting in leukemia (Hoggatt et al., 2016; Orkin and Zon, 2008). Understanding the intracellular transcriptional regulatory networks and signaling modulatory networks responsible for the equilibrium of HSCs is essential to controlling leukemogenesis and requires further investigation.

Lineage relations between HSCs, intermediate progenitors, and mature cells form a complex roadmap of transcriptional and epigenetic regulation for the developmental transitions, which are not fully understood. As intrinsic determinants of the cellular phenotype, TFs serve as an entry point for unraveling programmed lineage-restriction differentiation. Master TFs in different hematopoietic lineages have been discovered and are reviewed elsewhere (Iwasaki and Akashi, 2007; Kim and Bresnick, 2007; Rothenberg, 2014). TFs such as RUNX1, LMO2, GATA2, TEL/ETV6, and SCL/TAL1 are required for HSC identity or function. However, how these TFs cooperatively control downstream transcription and modulate the equilibrium between self-renewal and differentiation in HSCs, and how they are further modulated by epigenetic control to determine the cell state and developmental hierarchy, remains elusive.

Recently, high-throughput profiling of transcriptomes (Laurenti et al., 2013; Novershtern et al., 2011), DNA methylation (Ji et al., 2010), the dynamic chromatin state (Lara-Astiaso et al., 2014), accessibility (Corces et al., 2016) (Tables 1 and 2), proteomes (Cabezas-Wallscheid et al., 2014), and other factors has provided comprehensive information for network analysis to determine the transcriptional drivers during fate decision in hematopoietic lineage development. For example, cells representing the major types involved in hematopoiesis were sorted and characterized by ATAC-seq and RNA-seq. By using ATAC peaks and an enhancer catalog, the TF-binding motifs enriched in each cell type could be inferred. Moreover, by integrating ATAC-seq with RNA-seq of each cell population in the hematopoietic hierarchy, the chromatin accessibility landscapes (regulons) and transcriptomes could be profiled. Intriguingly, the chromatin accessibility is more cell-type specific in the major hematopoietic cell populations than mRNA expression, suggesting that the epigenomic features are better indicators than the transcriptome for capturing the hematopoietic identity of these cells. The TF regulatory network of normal hematopoiesis could be reconstructed based on footprinting analysis of ATAC-seq data. TFs such as GATA, RUNX, and SPI1 were found to be dominant regulators of chromatin accessibility. Activation of these TFs was cell-type specific, often displaying stepwise gains across developmental lineages. For example, GATA and PAX motifs are strongly enriched in erythroid and lymphoid lineages, respectively.

**Table 2:** Recent System-wide Epigenetic Datasets for Inferring Regulatory Networks of Hematopoiesis

		Description	Reference	Accession
Chromatin accessibility	*Mus musculus*	Spatial genome re-organization between fetal and adult hematopoietic stem cells (ATAC-seq).	Chen et al. (2019)	GSE119198
	*Homo sapiens*	ATAC-seq profiles of hematopoietic and leukemic cell types, across 13 normal hematopoietic cell types and three acute myeloid leukemia cell types. The complete dataset contains a total of 132 samples.	Corces et al. (2016)	GSE74912
		Single-cell ATAC-seq (scATAC-seq) of lymphoid-primed multipotential progenitors, monocytes, leukemic stem cells, and leukemic blast cells.	Corces et al. (2016)	GSE74310
		Single-cell epigenomics and transcriptomics mapping the continuous regulatory landscape of 10 cell types of human hematopoietic differentiation (bulk and single-cell ATAC-seq).	Buenrostro et al. (2018)	GSE96769, GSE96771
		Integrated epigenomic and transcriptomic profiling of terminal human erythropoiesis (bulk ATAC-seq).	Ludwig et al., 2019	GSE115672
ChIP-seq	*Mus musculus*	ChIP-seq of granulocyte-monocyte progenitor cells	Olsson et al., 2016	GSE70237
		Chromatin state dynamics during blood formation. The iChIP method was used to profile the chromatin dynamics during hematopoiesis across 16 different cell types, which include the principal hematopoietic progenitors.	Lara-Astiaso et al. (2014)	GSE59636
		ChIP-seq of Lin^−^CD34^+^ and Lin^−^CD34^−^ cells.	You et al., 2018	GSE100689
		Spatial genome re-organization between fetal and adult hematopoietic stem cells (ChIP-seq).	Chen et al. (2019)	GSE119200
Methylation	*Mus musculus*	The complete methylome of hematopoietic stem cells and their immediate progeny. Whole-genome bisulfite sequencing was performed on hematopoietic stem cells and their immediate progeny, namely, three different multipotent progenitor subpopulations (MPP, MPP1, and MPP2).	Cabezas-Wallscheid et al. (2014)	GSE52709

Systemic epigenetic profiling of mouse and human hematopoietic populations using ATAC-seq, ChIP-seq, and whole-genome bisulfite sequencing was collected from the literature and sorted into the table. The accession numbers from NCBI GEO were also listed. The datasets were found by searching the keywords “hematopoiesis” and “ATAC” or “ChIP” or “bisulfite” in NCBI GEO datasets portal uploaded in the previous 6 years and manually refined.

The stepwise gain in TF activation appears to support a paradigm shift from the classical hematopoietic development model that describes a progressive loss of differential potential as the cells move toward early fate commitment in the embryo stage (Laurenti and Göttgens, 2018). For example, a study of myeloid progenitor cells by massively parallel scRNA-seq (MARS-seq) has computationally identified diverse transcriptional networks in myeloid progenitors (Paul et al., 2015). Based on these identified transcriptional networks, myeloid progenitor cells are transcriptionally classified into at least seven different fates: erythrocytes, megakaryocytes, DCs, monocytes, neutrophils, eosinophils, and basophils. Moreover, no progenitors with mixed lineage states are observed under normal conditions, which further supports an early commitment model.

Although bulk transcriptome and chromatin accessibility profiling of purified cell types are common strategies to study the transcriptional regulatory networks in hematopoiesis, the functional heterogeneity within the identified populations might have been underestimated. To overcome this limitation, several studies have used the emerging single-cell technologies, which have enabled molecular heterogeneity to be explored systematically during hematopoiesis (Giladi et al., 2018; Notta et al., 2016; Velten et al., 2017). scATAC-seq was performed to examine the regulons and infer the TF activity with single-cell resolution (Buenrostro et al., 2018), and strong heterogeneity was identified within populations of common myeloid progenitor and granulocyte-macrophage progenitor cells. Similar to bulk ATAC-seq, scATAC-seq data could also be integrated with scRNA-seq data to associate changes in TF expression with the changes in chromatin accessibility at *cis*-regulatory elements and to explore the dynamic gene-regulatory elements. Specifically, the analysis yielded 11 TFs that defined different stages of myeloid development, including the loss of expression of HOX factors and the activation of SPI1 (PU.1) and IRF8. Thus, studies of single-cell chromatin accessibility, coupled with single-cell transcriptomics, have helped to decipher the tight regulation of selective expression of TFs along the hematopoietic trajectory, adding another layer of transcriptional and epigenetic regulation of early commitment.

In addition to being orchestrated by intracellular transcriptional regulatory networks during hematopoiesis, the differentiation of hematopoietic cells is modulated by their developmental microenvironments, mainly bone marrow niches where distinct mesenchymal cells, the vasculature, and differentiated hematopoietic cells interact to regulate the maintenance and differentiation of HSCs (Morrison and Scadden, 2014). Although classical studies using genetic approaches have yielded important insights into the functional roles and cellular sources of key cytokines, such as Cxcl12 and Scf (*Kitlg*), during hematopoiesis, the limitations of studying a potential heterogeneous population by using single markers and the lack of systemic investigation of intercellular interactions between HSCs and their interacting cells have prompted debate about the importance of distinct cell types and the corresponding ligand-receptor pairs (Asada et al., 2017; Ding and Morrison, 2013; Ding et al., 2012; Greenbaum et al., 2013; Kunisaki et al., 2013; Méndez-Ferrer et al., 2010; Sugiyama et al., 2006). The expression of ligands and receptors, which serve as the bridge between two communicating cells, has been examined in the bone marrow microenvironment in recent scRNA-seq studies (Baryawno et al., 2019; Tikhonova et al., 2019). One study combined scRNA-seq and spatial transcriptomics to reveal the cellular and spatial organization of the bone marrow niche and the cell-cell communication network (Baccin et al., 2020). The RNA-Magnet algorithm, based on the expression of ligands and receptors, was used to infer the intercellular communication at different niches in bone marrow, including sinusoidal, endosteal, and arteriolar niches. The results demonstrated that CXCL12-abundant reticular (CAR) cell subsets (adipo-CAR and osteo-CAR cells) differentially localize to sinusoidal and arteriolar surfaces, act locally as “professional cytokine-secreting cells,” and establish perivascular niches for hematopoietic progenitor cell development. Other methods, such as ProximID, that use mild microdissection can also characterize the intercellular network while preserving bone marrow niches (Boisset et al., 2018). Besides bone marrow, the thymus is also an important microenvironment in which immature T lymphocytes develop into mature cells. Park and colleagues have recently generated a cell atlas of human thymic development that defines T cell repertoire formation (Park et al., 2020). Similar to bone marrow niches, thymic stroma cells play a critical role in instructing T cell maturation (Stritesky et al., 2012). CellPhoneDB was used to investigate cellular interactions between these cells. The predicted results suggest that stromal cells recruit ILC3 by CXCL13:CXCR5 interaction and induce thymic epithelial cell development by FGF7:FGFR2 signaling. Furthermore, multiple stromal cells express different Notch ligands to interact with and instruct early thymocyte progenitors by binding with the NOTCH1 receptor. These intercellular network analyses demonstrate that specific combinatorial signaling input from different local niches is essential for hematopoiesis. Whether the newly found ligand-receptor interactions determine the early fate decisions of HSC or T lymphocyte maturation is an exciting question to investigate.

The major types of blood cells generated by hematopoiesis, such as monocytes, macrophages, and lymphoid cells, require further differentiation to become tissue-specific compartments for specialized functions (Bassler et al., 2019; Wong et al., 2016). For instance, microglia are macrophages that reside in the central nervous system (CNS), where they are constantly scavenging for plaques, damaged or unnecessary neurons and synapses, and infectious agents (Li and Barres, 2018). Enormous progress has been made in understanding the ontogeny of tissue-specific myeloid cells. Different tissue-specific macrophages have been found to play indispensable roles in tissues such as the brain (Paolicelli et al., 2011), spleen red pulp (Chow et al., 2013; Kohyama et al., 2009), and peritoneum (Okabe and Medzhitov, 2014). Most tissue-specific macrophages are established prenatally from embryonic precursors and are maintained by self-renewal independently of monocytes under homeostatic conditions (Ginhoux et al., 2010; Gomez Perdiguero et al., 2015; Hashimoto et al., 2013; Hoeffel et al., 2015; Schulz et al., 2012; Sheng et al., 2015; Yona et al., 2013). However, macrophages also exhibit distinct phenotypes in response to microenvironmental factors (Butovsky et al., 2014; Haldar et al., 2014; Okabe and Medzhitov, 2014). Therefore, understanding the mechanisms underlying macrophage ontogeny requires sophisticated approaches such as network-based methods to uncover the molecular networks orchestrating tissue-specific macrophage development. For example, the tissue-specific macrophages that undergo self-renewal activate a series of genes that are also required for embryonic stem cell self-renewal (Soucie et al., 2016). These “self-renewal” genes could be constructed into a self-renewal network based on their cross-regulation as observed by short hairpin RNA silencing experiments. To explore how microenvironmental factors contribute to distinct phenotypes in tissue-resident macrophages, two groups systematically assessed differences in the chromatin state and gene expression in macrophages isolated from tissues, using RNA-seq, ChIP-seq, and ATAC-seq to elucidate the transcriptional and epigenomic networks (Gosselin et al., 2014; Lavin et al., 2014). Distinct enhancer landscapes that reflect the microenvironment were found, and a computational pipeline was established to identify candidate TF regulators for each enhancer cluster. The results indicated that distinct enhancer landscapes of tissue-resident macrophages result from the restricted expression and binding of TFs. The studies also suggested that the tissue microenvironment could cross talk with tissue-restricted TFs in conjunction with those ubiquitously expressed in macrophages, such as PU.1, to orchestrate tissue-specific macrophage development. For example, Gata6 and Mef2 are likely regulators of peritoneal and microglia-specific macrophage enhancers, respectively. Other TFs, including Lxra in Kupffer cells (liver macrophages), Pparγ in lung macrophages, and Runx3 in intestinal macrophages, are responsible for the enhancer landscape in each tissue.

In contrast to their behavior in normal homeostasis, under pathological conditions, monocytes infiltrate affected tissues and differentiate into macrophages to support efficient resolution of the inflammation (Epelman et al., 2014). How intracellular transcriptional regulatory and signaling networks control the differentiation of monocytes to macrophages in various tissues awaits more systematic investigation. These gene regulatory network-based studies also demonstrated one limitation of using surface markers to define the myeloid progenitors and revealed more heterogeneity in these progenitor cells. Moreover, a revised model of biased hematopoietic differentiation may be required because the loss of self-renewal and stepwise progression through specific differentiation stages are not essential for lineage commitment (Sanjuan-Pla et al., 2013; Yamamoto et al., 2013).

### Characterization of Intracellular and Intercellular Networks Mediating T cell Activation/Differentiation in Adaptive Immunity

A fundamental goal in adaptive immunity is to characterize the activation and differentiation of T lymphocytes, which mainly comprise CD4^+^ and CD8^+^ T cells, during an immune response (Taniuchi, 2018). The process is capable of attaining distinct effector or regulatory states in different inflammatory milieus, such as developing effector and memory T cells to curtail infections, and is orchestrated by TFs, signaling proteins, and their downstream target genes, as well as by related chromatin changes and microRNAs (Singh et al., 2014) in a network framework. Recent advances in technologies for transcriptome- and proteome-scale measurements and perturbations have provided a tremendous opportunity for systemically understanding T cell activation and differentiation (Amit et al., 2011). One application with great potential is the unbiased reconstruction of genetic and molecular networks. In such studies, inferring the activity of the TFs from their downstream targets and investigating the co-expression pattern of genes/proteins based on profiling the global scale of dynamic changes in the transcriptome or proteome are widely used. These studies have led to a deeper understanding of the specific components in such networks. Here we summarize some case studies to demonstrate how network methods were applied.

Naive T cells exist in a quiescent state characterized by small cell size and no active cell cycle (Chapman et al., 2020). Upon antigen stimulation, the engagement of T cell receptors triggers a signaling cascade to initiate cell growth and proliferation and, ultimately, differentiation into effector cells. However, the transitional process by which naive T cells exit quiescence remains poorly understood. Deep proteomics profiling provides an opportunity to characterize the proteome dynamics during T cell activation. Tan et al. (2017) profiled the whole proteome and phosphoproteome via multiplexed isobaric labeling proteomics technology to dissect the regulatory networks underlying T cell activation. Specifically, WGCNA for differentially expressed proteins during T cell activation was first performed to identify clusters of highly correlated proteins (Jansen et al., 2002). The identified proteins in each cluster were then superimposed on the PPI network to identify functional modules. Furthermore, by integrating proteome and phosphoproteome profiling with known TF-target and kinase-substrate databases, the connectivity between TFs and kinases was constructed. The unbiased analysis identified specific TFs and kinases and their interplays in mediating transcriptional, translational, and post-translational control of T cell activation. Besides the computational inference of molecular circuits from whole-proteome profiling, quantitative interaction proteomics was developed to characterize the protein interaction network in primary T cells directly (Shi et al., 2018, 2019; Voisinne et al., 2019). Studies have been conducted to construct the T cell receptor proximal signaling network through quantitative interaction proteomics (Voisinne et al., 2019), which also contribute to our understanding of T cell exit from quiescence.

**Figure 4:**
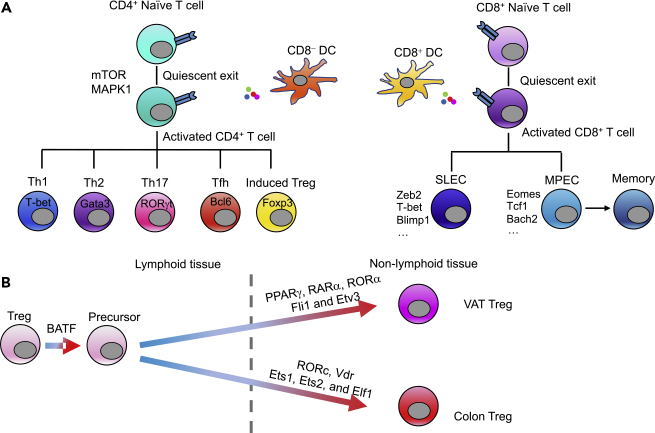
T cell Polarization and Differentiation and Nonlymphoid Treg Cell Generation (A) T cell polarization into different specialized cell states is crucial for the mammalian immune response. The process is tightly controlled by the cytokines and other molecules that are present when T cells are activated by antigen-MHC (major histocompatibility complex) complexes, in which dendritic cells (DCs) play a critical role. CD8α^+^ DCs exhibit a preference for priming CD8^+^ T cells over CD4^+^ T cells, whereas CD8α^−^ DCs are more efficient at priming CD4^+^ T cells. Specifically, CD4^+^ naive T cells undergo quiescent exit during activation and differentiate into Th1, Th2, Th17, Tfh, or induced regulatory T (Treg) cells based on the cytokines present in the microenvironment to help control the magnitude of the immune response. Several transcription factors (TFs), such as T-bet for Th1, Gata3 for Th2, RORγt for Th17, Bcl6 for Tfh, and Foxp3 for induced Treg cells, are critical for this differentiation. In contrast, CD8^+^ naive T cells will become activated upon being presented with a specific antigen and differentiate into a heterogeneous pool of effector T cells that consists of two major subsets: short-lived effector cells (SLECs) and memory precursor effector cells (MPECs). Most SLECs die by apoptosis during the contraction phase, whereas MPECs survive and become long-lived memory cells. TFs such as Zeb2, T-bet, and Blimp1 are critical for SLEC generation, whereas TFs such as Eomes, Tcf1, and Bach2 are necessary for MPEC differentiation. (B) Treg cells display tissue-specific heterogeneity, enabling them to not only suppress an inflammatory response but also play an essential role in tissue homeostasis. Recent studies have identified a developmental trajectory of Treg cells from lymphoid tissue to nonlymphoid tissue. Precursors of nonlymphoid tissue Treg cells reside in lymphoid tissue and are programmed by the transcription factor BATF. Gene regulatory network-based analysis has identified three nuclear receptor family TFs, Pparγ, Rara, and Rora, and two ETS family TFs, Fli1 and Etv3, as being specifically associated with visceral adipose tissue (VAT)-specific genes and VAT-Treg cell development, whereas the TFs Rorc, Vdr, Ets1, Ets2, and Elf1 have been linked specifically to colon-specific genes and the generation of colon-specific Treg cells.

Activated CD4^+^ T cells further differentiate into different T helper cells (e.g., Th1, Th2, Th17, and Tfh cells) or induced Treg cells to exert various immune functions (Taniuchi, 2018) (Figure 4A). Among them, pro-inflammatory IL-17-producing Th17 cells can mediate the clearance of pathogen infections (Hernández-Santos and Gaffen, 2012) and the pathogenesis of autoimmunity (Korn et al., 2009). Consistent with their functional diversity, Th17 cells are characterized by inherent plasticity (Hirota et al., 2011, 2013; Lee et al., 2009, 2012). The scRNA-seq technology has been applied to dissect intrinsic circuits underlying this plasticity under different immunologic conditions. Perturbations derived from changes in the microenvironment or from genetic alterations could lead to a different transcriptional landscape in Th17 cells. For example, Th17 cells isolated from the draining lymph nodes and CNS at the peak of experimental autoimmune encephalomyelitis (EAE) exhibited diverse functional states in principal-components analysis (Gaublomme et al., 2015). TFs whose targets (regulons) were strongly enriched in major principal components were identified to explain the heterogeneity and progress from lymph nodes to the CNS during disease development. Co-variation analysis was also performed on pro-inflammatory and regulatory modules in Th17 cells to construct the network, highlighting novel regulators such as Gpr65 for Th17 cell pathogenicity in EAE. Another study examined the activity of transcriptional factors in wild-type and Raptor (a major component of the mTOR protein complex)-deficient Th17 cells by using Ingenuity Pathway Analysis based on downstream target-gene expression (Karmaus et al., 2019). The altered TF landscape of the transcriptional regulatory network in Raptor-deficient Th17 cells could also be validated by examining the chromatin accessibility via ATAC-seq (Gabryšová et al., 2018). Moreover, a comprehensive study has been performed to compare the chromatin accessibility of Th17 cells with that of other types of T helper cell to construct transcriptional regulatory networks in Th17 cells (Miraldi et al., 2019). This study refined and extended the Th17 transcriptional regulatory networks by using an inference method named mLASSO-StARS to integrate all Th17 data (gene expression, ATAC-seq, TF knockout, and ChIP-seq data). “Core” Th17 transcriptional regulatory networks were identified, including well-known TFs (e.g., Rorc, Rora, and Stat3) and novel ones. Unbiased genome-wide association study analysis also associated new phenotypes with TFs in the constructed Th17 network, such as the function of FOXB1 in regulating inflammatory bowel disease-related genes.

Other network analysis algorithms, such as ISMARA, have also been used to study the differentiation of other T helper cells. Henriksson and colleagues have built a network by linking TFs to potential target genes based on the presence of the relevant motif in an ATAC-seq peak in the vicinity of the transcription start site of a target gene (Henriksson et al., 2019). TFs were categorized according to their activity over the time course of Th2 differentiation and whether their activity differed between Th2 cells and Th0 cells (activated but not differentiated T cells). The MARA approach could extract canonical Th2 TFs, such as Stat6, Gata3, and Batf, and highlighted TF hits (E2f1 and Foxo1) that are also likely to be relevant for Th2 development. These network-inferred TFs could be further functionally validated by CRISPR-Cas9-mediated gene deletion during Th cell differentiation.

Aside from conventional helper and effector T cells that develop during inflammation, tissue-resident T cells display distinct phenotypes, and this is important for maintaining homeostasis and immune tolerance in the tissue microenvironment. For example, it has become increasingly clear that CD4^+^Foxp3^+^ Treg cells accumulate in various non-lymphoid tissues, where they exert both anti-inflammatory and homeostatic functions. Certain critical elements, such as the molecular factors and transcriptional network that drive tissue-specific Treg cell generation, remain elusive. Recent studies have integrated ATAC-seq and scRNA-seq data to elaborate the transcriptional networks underlying the unique tissue-specific Treg transcriptomes in adipose tissues, muscle, and colon (DiSpirito et al., 2018). TF families are first linked to target genes by ATAC-seq; then the co-variation in expression between a TF and its putative targets is calculated to determine whether the putative controlling factors could be functional. To generate a network, the tissue-specific TFs within each family were identified, and connections with significant correlations between the TFs and target genes in the scRNA-seq data were retained. Based on the generated gene regulatory network, nuclear receptor family TFs, including Pparγ, Rara, and Rora, were specifically associated with VAT-specific genes, whereas Rorc and Vdr were linked specifically to colon-specific genes. The ETS-family TFs Fli1 and Etv3 partitioned to VAT-specific loci, whereas Ets1, Ets2, and Elf1 partitioned to colon-specific loci (Figure 4B). Further network analysis revealed that tissue-restricted and broadly acting TFs were integrated into feed-forward loops to enforce tissue-specific gene expression in non-lymphoid tissue Treg cells. This study provides a network view of the epigenetic dynamics of tissue-specific Treg cells that could be applied to other immune cells in the future.

Antigen-presenting cells, especially DCs, are critical for presenting antigens to T cells and inducing T cell activation. Among the different subtypes of DCs, CD8^+^ DCs have superior ability to prime CD8^+^ T cell activation, whereas CD8^−^ DCs are more efficient at priming CD4^+^ T cell activation during an immune response (Dudziak et al., 2007). To understand the intracellular signaling network that is responsible for DC-mediated T cell priming, SJARACNe (Khatamian et al., 2019) and NetBID (Du et al., 2018) have been successfully applied by integrating data from transcriptomics, whole proteomics, and phosphoproteomics. The inferred signaling network shows that multiple Hippo signaling proteins are enriched in the CD8^+^ DCs. These results were validated by confirming the phosphorylation of the Hippo signaling kinases, which indicates their activation. Intriguingly, because the inference of putative drivers is based on their regulons, drivers with no expression change could be identified, showing the advantages of this approach over common differential gene expression analysis. Indeed, the core kinase of Hippo signaling, Mst1, was identified as a critical driver for CD8^+^ DCs, when compared with CD8^−^ DCs, with no obvious change in protein expression.

The intercellular communication network between DCs and T cells for T helper cell differentiation was also assessed. In one recent study, 36 DC-derived ligand signals and 17 Th cytokines broadly covering Th diversity were measured to develop a data-driven, computationally validated model to identify potentially novel intercellular mechanisms of Th cell specification (Grandclaudon et al., 2019). This study has provided a resource for deciphering the combinatorial rules of ligand-receptor interactions between DCs and T cells. The recently developed PIC-seq method also detected increased expression of ligands and receptors in DC-T cell conjugates that could drive their interaction (Giladi et al., 2020). Notably, Rieckmann and colleagues performed high-resolution MS-based proteomics to characterize systematically the intercellular communications among 28 primary human immune cell populations in the steady and activated states (Rieckmann et al., 2017). After activation, DCs showed an increase in their ligand:receptor ratio, whereas the corresponding ratio for cytolytic CD8^+^ T cells decreased. In brief, DCs and T cells employed distinct communication strategies: DCs changed both the quantity and diversity of their ligands and reduced their receptor diversity, whereas cytolytic CD8^+^ T cells increased their receptor diversity. These results highlight the importance of the intercellular network between DCs and T cells in instructing T cell activation and function.

In summary, the intracellular transcriptional regulatory and signaling networks responsible for T cell activation, differentiation, and tissue adaption, as well as the intercellular network that regulates DC-mediated T cell differentiation, are intricate, dynamic, multilayered, and context dependent. The driver TFs and ligand-receptor pairs identified in studies of intracellular modulation and intercellular communication, respectively, could be used to select candidate regulators and a representative signature of the output for perturbation studies. The recently developed techniques based on large-scale CRISPR-based perturbation in cells coupled with scRNA-seq, such as Perturb-seq, CRISPR-seq, and CROP-seq, have been used to study the response of immune cells to pathogen or antigen stimulation (Datlinger et al., 2017; Dixit et al., 2016; Jaitin et al., 2016). These technologies enable analysis of molecular networks in the response to multiple genetic perturbations in primary T cells at the single-cell level. A combinational role of TFs and/or ligand-receptor pairs on T cell activation, differentiation, and tissue adaption could thereby be unveiled, further refining the network.

### Exploration of Gene Regulatory Network in B Cell Differentiation and Humoral Immunity

Similar to their T cell counterpart, B cells have a penetrating influence on adaptive immunity, especially on humoral immune responses (Akkaya et al., 2020; Cyster and Allen, 2019). Antibody is produced by small populations of terminally differentiated B cells, known as plasmablasts (plasma cell precursors) and plasma cells (Nutt et al., 2015). Following the detection of foreign antigens, the differentiation of B cells into antibody-secreting plasma cells is driven by dramatic alternations to the transcriptional programs, including the concerted action of several TFs and epigenetic regulators (Willis and Nutt, 2019). Although the study of B cell differentiation is a mature field with key events within the scheme well known, the distinct transcriptome, especially the transcriptional regulators, between plasma cells and follicular B cells have implied complex gene regulatory network changes during the differentiation (Shi et al., 2015).

Several network-based methods have been applied to understand the way B cells integrate upstream signals to control the differentiation. Regulatory network modeling is a valuable approach to describe the biological process that lacks detailed kinetic information. Regulatory network controlling the terminal differentiation of B cells can be inferred from the experimental data available in the literature regarding the key molecules found during the differentiation, including TFs and external stimuli like antigens and cytokines (Mendez and Mendoza, 2016). By comparing with known stationary molecular patterns in different stages of differentiating B cells, a dynamic model was constructed to describe the cellular differentiation pattern under various external signals and predict molecular interactions and intermediate states that are necessary for the terminal differentiation. By incorporating transcriptome data, WGCNA were also used to define novel coregulated gene modules in plasmablast to plasma cell transition upon external stimuli. For instance, type I IFN has been identified to enhance the generation of plasma cells both *in vivo* and *in vitro* (Cocco et al., 2012; Jego et al., 2003). WGCNA was able to identify the co-expressed gene modules responsible for IFN stimulation in plasmablasts and construct gene regulatory network to define inflammatory regulatory circuits during plasma cell differentiation (Care et al., 2016). Other gene correlation-based methods such as parsimonious gene correlation network analysis were also used to assess the gene regulatory network underlying the transition from activated B cells toward plasmablasts (Cocco et al., 2019).

However, understanding the correlation between genes expression is not enough to find the upstream drivers in the network. The establishment of transcriptional programs by gene regulatory network in differentiating B cells requires not only the *trans*-acting factors like TFs and their co-factors but also *cis*-acting regulatory DNA elements such as enhancers and promoters. With the help of recently developed technologies like chromatin accessibility by formaldehyde-assisted isolation of regulatory elements and sequencing (FAIRE-seq) (Gaulton et al., 2010) and ATAC-seq, enhancer fragments can be detected, further cloned into plasmid vectors as a library, and transfected into activated B cells using self-transcribing active regulatory region sequencing (STARR-seq) to assess “active” enhancer binding (Chaudhri et al., 2020). Such coupled approaches allow detecting functional active regions and depict the gene regulatory network (*cis*-regulome) that modulates B cell differentiation. For example, genomic chromatin occupancy of B cell lineage-determining TFs and motif enrichments reveal that enhancers can be poised by the binding of lineage TFs (including Ebf1, Pax5, E2A, PU.1, Ets1, and Blimp-1), but that enhancer activation is associated with extensive collaborative binding of additional signal-responsive TFs such as NF-κB and IRF4. Other integrative network analysis such as coupling ATAC-seq with RNA-seq or bisulfite-seq could also help to reveal the correlation between motif accessibility and TF gene expression or *de novo* DNA methylation (Barwick et al., 2018; Scharer et al., 2018), which is critical to understand the whole landscape of active transcriptional and epigenetic control during B cell activation and the plasma cell differentiation. Hence, it is possible to incorporate phenotypes and technologies from other immunological fields into the complex developmental schemes that unfold B cell activation and antibody production. With the prevailing single cell-based technologies, cell-type specific gene regulatory network of B cells in different contexts, such as cancer (Helmink et al., 2020), would be a future direction to explore.

### Inference of Network Regulation in the Tumor Microenvironment

Immune cells play a critical role in modulating anti-tumor responses in the tumor microenvironment (TME) (Binnewies et al., 2018; Gajewski et al., 2013). The TME is an unusual tissue-specific milieu for immune cells, wherein the existence of various immune cells can be a barrier or an aid to tumor progression. For example, effector CD8 T cells attack and induce the death of tumor cells, whereas Treg cells can suppress the anti-tumor response. Tumor cells can escape immune detection by upregulating the expression of ligands for immune checkpoint molecules such as PD-1 (Binnewies et al., 2018). Therefore, immunotherapies such as anti-PD1 antibody treatment have been developed to boost the effector function of immune cells (Topalian et al., 2015). However, not all tumors or patients respond to these immune checkpoint-based therapies, indicating the heterogeneity of the intricate tumor immune ecosystem. Therefore, we face an enormous challenge in attempting to understand how different cell types, cell states, and intercellular communications remodel the microenvironment and ultimately shape therapeutic responses and resistance. The recent advances in single-cell sequencing technologies have provided us with a powerful approach to dissecting the heterogeneity in the TME. How to best use the large amount of single-cell data to reconstruct the intracellular signaling networks and extracellular communication networks requires further consideration.

**Figure 5:**
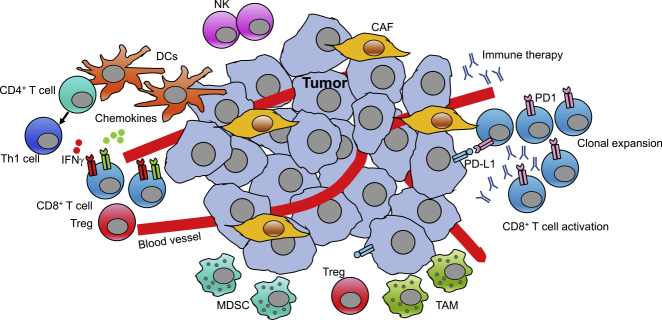
The Immune Landscape and Intercellular Communication Network in the Tumor Microenvironment (TME) The TME consists of a complex and heterogeneous population of multiple cell types of different origins that are divided into two major groups: tumor cells and stromal cells. The stromal cells comprise cancer-associated fibroblasts (CAFs), endothelial cells, and immune cells. The immune cells are also heterogeneous and participate in pro- or anti-tumorigenic activities that are influenced by the surrounding signaling. For instance, Treg cells, myeloid-derived suppressor cells (MDSCs), and tumor-associated macrophages (TAMs) are immunosuppressive and pro-tumorigenic, whereas CD8^+^ T cells, Th1 cells (facilitated by dendritic cell [DC]-mediated generation), and natural killer (NK) cells increase anti-tumor responses. Moreover, the implications of tumor and immune cell intercellular interactions, such as those involving PD-L1/PD1, have led to the development of immune therapies such as targeting PD1 on CD8^+^ T cells, thus inducing activation and clonal expansion of tumor-reactive CD8^+^ T cells.

Unlike normal tissues, the tumor environment is composed of a pool of immune cells, cancer-associated fibroblasts (CAFs), and malignant cells (Figure 5). Dissecting the gene regulatory network for each cell type could infer modulators of tumor progression. For example, the innate immune cells, including tumor-associated macrophages (TAMs), play important but sometimes opposing roles in tumor progression. Various stimuli can lead to a range of innate immune cell states, resulting in heterogeneous subpopulations, such as M1- and M2-polarized TAM classes. M1 polarization is more anti-tumor, whereas M2 polarization is more pro-tumor. In this regard, a holistic picture of the complexity of the molecular circuits is required to characterize innate immune cell polarization. By literature mining, the molecular mechanism governing the innate immune response in cancer was presented as a comprehensive network map (Kondratova et al., 2019). Functional modules were identified in this network map, and the anti-tumor and pro-tumor phenotypes of innate immune cells could be calculated based on the functional module activity in different tumor contexts, which could potentially serve as a source of a patient survival signature.

Despite the comprehensiveness of the literature-based network map, context-specific changes in the TME may not be captured. Tumor-specific transcriptional networks still need to be constructed to infer the molecular circuits responsible for tumor progression. For instance, to contextualize systematically both cellular and molecular shifts in the cascade from gastric premalignant lesions to early gastric cancer, a single-cell transcriptome network was constructed by assessing the similarity of each pair of cell types and connecting marker genes for each representative cell type in each lesion based on known PPIs documented in the STRING database (Zhang et al., 2019a). In this case, the signature genes and the corresponding regulatory network contributing to tumor progression could be identified. A similar study leading to the construction of a gene regulatory network was conducted in immune cells (Clancy and Hovig, 2016). The signature genes of immune cells in the tumor environment were used to query the PPI network in an effort to capture the gene regulatory networks in immune cells that are responsible for tumor progression. However, common PPI networks are usually obtained from general databases, mostly likely originating from cell lines. In future, a cell-type-specific PPI network is to be preferred for better integration with scRNA-seq data.

Understanding the intratumor heterogeneity of immune cells is critical. Similar to a social network, immune response in tumors also involve various cell types. The main actors in such a social network are the immune cells and tumor cells, and the interactions are the communications through adhesion molecules, cell-surface signaling receptors, and secreted proteins, such as cytokines and chemokines. The identification of the interaction between tumors and immune cells has led to the discovery of immune checkpoint therapies. Recent successes in targeting molecules such as PD-1/PD-L1 and CTLA-4 have prompted detailed investigations of other intercellular ligand-receptor pairs. Because traditional immune checkpoint therapies have durable effects in only a minority of patients and are prone to selectivity for tumor types, novel forms of tumor-specific intercellular communication await systematic investigation. The Cancer Genome Atlas (TCGA) has profoundly illuminated the genomic landscape of human malignant neoplasms. Transcriptomic data from bulk tumor samples can be used to study the tumor environment. One comprehensive study used TCGA data compiled from more than 10,100 tumors comprising 33 diverse cancer types to dissect the extracellular networks (Thorsson et al., 2018). Immune cells such as CD4^+^ and CD8^+^ T cells were inferred by CIBERSORT, a method for characterizing the cell composition of complex tissues based on their gene expression profiles. A network of documented ligand-receptor, cell-receptor, and cell-ligand pairs was retrieved from the FANTOM5 resource and augmented with the additional interactions identified. The integrative network yielded a holistic picture of specific modes of extracellular control among immune cells in the TME; highlighted the role of key receptors and ligands, such as TGFB1, CXCL10, and CXCR3, and ligand-receptor pairs, such as CCL5-CCR5; and illustrated how immune cell interactions may differ depending on the environmental context.

The rapidly burgeoning use of single cell-based transcriptome analysis has enabled a higher resolution of the ligand-receptor pairs in the TME. One scRNA-seq study in samples from patients with melanoma identified a set of genes expressed in CAFs, such as those encoding complement proteins, whose expression correlated strongly with T cell infiltration; this implies that complement activity of CAFs is important for T cell recruitment and for modulating T cell-mediated anti-tumor immune responses (Markiewski et al., 2008; Tirosh et al., 2016). Further studies used a set of immune-related ligand-receptor pairs from the literature (Chen and Flies, 2013; Ramilowski et al., 2015; Szklarczyk et al., 2015; Vento-Tormo et al., 2018) and examined their expression to gain insights into the regulatory relations among the cell clusters identified (Kumar et al., 2018; Yuan et al., 2019; Zhang et al., 2019b). The ligand-receptor pairs were then examined for their correlation with patient survival to uncover their contribution to tumor progression. To characterize ligand-receptor interactions more accurately, one must incorporate the spatial information, because whether two cells indeed interact with each other largely depends upon their locations. A simple experimental approach to validate the interactions is to use *in situ* methods such as immunohistochemical staining to confirm the proximity of the cells (Zhang et al., 2019b). Moreover, recently emerged spatial transcriptomics methods provide another layer of spatial information along with the expression data, which could increase the accuracy of the ligand-receptor inference for a pair of cells (Eng et al., 2019; Rodriques et al., 2019). Machine learning-based inference of cellular compartments would also assist in systematically depicting and constructing a special network of the extracellular interactions in the TME. These integrative methods should shed light upon the extracellular signals involved in instructing the immune cells in their migration, activation, and pro- or anti-tumor functions and should provide new targets for next-generation immune therapy.

## Concluding Remarks and Future Perspectives

The complexity of the immune system makes it particularly well suited to the application of network analysis. Despite the recent advances in high-throughput omics technologies and computational methodologies, we are still far from achieving the eventual goal of applying network-based approaches to predict immune responses. There are a few major challenges in the field. First, although different kinds of molecular networks are computationally inferred separately, they function together via complex interplay. Recently, advances in single-cell technology have enabled multimodal omics measurements in which various layers of genomic information are captured simultaneously (Efremova and Teichmann, 2020; Zhu et al., 2020). However, measuring information across different location and timescales, such as both gene and protein expression, or both intracellular and intercellular interactions, is more challenging. Another challenge is modeling the network dynamics. Currently, dynamic modeling is possible only for systems with a dozen or so components; modeling is rather limited in the genome-wide context, partly because tracking many components in real time is still quite a formidable undertaking. Nevertheless, as omics technologies continue to be revolutionized, we expect to see a new generation of network algorithms coming into play.

## Methods

All methods can be found in the accompanying Transparent Methods supplemental file.

## References

[bib1] Abkowitz J.L., Catlin S.N., McCallie M.T., Guttorp P. (2002). Evidence that the number of hematopoietic stem cells per animal is conserved in mammals. Blood.

[bib2] Adlung L., Amit I. (2018). From the Human Cell Atlas to dynamic immune maps in human disease. Nat. Rev. Immunol..

[bib3] Aebersold R., Mann M. (2016). Mass-spectrometric exploration of proteome structure and function. Nature.

[bib4] Aibar S., González-Blas C.B., Moerman T., Huynh-Thu V.A., Imrichova H., Hulselmans G., Rambow F., Marine J.C., Geurts P., Aerts J. (2017). SCENIC: single-cell regulatory network inference and clustering. Nat. Methods.

[bib5] Akkaya M., Kwak K., Pierce S.K. (2020). B cell memory: building two walls of protection against pathogens. Nat. Rev. Immunol..

[bib6] Alvarez M.J., Shen Y., Giorgi F.M., Lachmann A., Ding B.B., Ye B.H., Califano A. (2016). Functional characterization of somatic mutations in cancer using network-based inference of protein activity. Nat. Genet..

[bib7] Amit I., Regev A., Hacohen N. (2011). Strategies to discover regulatory circuits of the mammalian immune system. Nat. Rev. Immunol..

[bib8] Andrews T.S., Hemberg M. (2018). Identifying cell populations with scRNASeq. Mol. Aspects Med..

[bib9] Arenas A., Fernández A., Gómez S. (2008). Analysis of the structure of complex networks at different resolution levels. New J. Phys..

[bib10] Asada N., Kunisaki Y., Pierce H., Wang Z., Fernandez N.F., Birbrair A., Ma'ayan A., Frenette P.S. (2017). Differential cytokine contributions of perivascular haematopoietic stem cell niches. Nat. Cell Biol..

[bib11] Baccin C., Al-Sabah J., Velten L., Helbling P.M., Grunschläger F., Hernández-Malmierca P., Nombela-Arrieta C., Steinmetz L.M., Trumpp A., Haas S. (2020). Combined single-cell and spatial transcriptomics reveal the molecular, cellular and spatial bone marrow niche organization. Nat. Cell Biol..

[bib12] Barabasi A.L., Oltvai Z.N. (2004). Network biology: understanding the cell's functional organization. Nat. Rev. Genet..

[bib13] Barwick B.G., Scharer C.D., Martinez R.J., Price M.J., Wein A.N., Haines R.R., Bally A.P.R., Kohlmeier J.E., Boss J.M. (2018). B cell activation and plasma cell differentiation are inhibited by de novo DNA methylation. Nat. Commun..

[bib14] Baryawno N., Przybylski D., Kowalczyk M.S., Kfoury Y., Severe N., Gustafsson K., Kokkaliaris K.D., Mercier F., Tabaka M., Hofree M. (2019). A cellular taxonomy of the bone marrow stroma in homeostasis and leukemia. Cell.

[bib15] Bassler K., Schulte-Schrepping J., Warnat-Herresthal S., Aschenbrenner A.C., Schultze J.L. (2019). The myeloid cell compartment-cell by cell. Annu. Rev. Immunol..

[bib16] Bhattacharya S., Andorf S., Gomes L., Dunn P., Schaefer H., Pontius J., Berger P., Desborough V., Smith T., Campbell J. (2014). ImmPort: disseminating data to the public for the future of immunology. Immunol. Res..

[bib17] Binnewies M., Roberts E.W., Kersten K., Chan V., Fearon D.F., Merad M., Coussens L.M., Gabrilovich D.I., Ostrand-Rosenberg S., Hedrick C.C. (2018). Understanding the tumor immune microenvironment (TIME) for effective therapy. Nat. Med..

[bib18] Blondel V.D., Guillaume J.-L., Lambiotte R., Lefebvre E. (2008). Fast unfolding of communities in large networks. J. Stat. Mech. Theor. Exp..

[bib19] Boisset J.C., Vivié J., Grün D., Muraro M.J., Lyubimova A., van Oudenaarden A. (2018). Mapping the physical network of cellular interactions. Nat. Methods.

[bib20] Bonnardel J., T'Jonck W., Gaublomme D., Browaeys R., Scott C.L., Martens L., Vanneste B., De Prijck S., Nedospasov S.A., Kremer A. (2019). Stellate cells, hepatocytes, and endothelial cells imprint the kupffer cell identity on monocytes colonizing the liver macrophage niche. Immunity.

[bib21] Boutros M., Ahringer J. (2008). The art and design of genetic screens: RNA interference. Nat. Rev. Genet..

[bib22] Bravo González-Blas C., Minnoye L., Papasokrati D., Aibar S., Hulselmans G., Christiaens V., Davie K., Wouters J., Aerts S. (2019). cisTopic: cis-regulatory topic modeling on single-cell ATAC-seq data. Nat. Methods.

[bib23] Brohée S., Faust K., Lima-Mendez G., Vanderstocken G., van Helden J. (2008). Network analysis tools: from biological networks to clusters and pathways. Nat. Protoc..

[bib24] Browaeys R., Saelens W., Saeys Y. (2020). NicheNet: modeling intercellular communication by linking ligands to target genes. Nat. Methods.

[bib25] Buenrostro J.D., Corces M.R., Lareau C.A., Wu B., Schep A.N., Aryee M.J., Majeti R., Chang H.Y., Greenleaf W.J. (2018). Integrated single-cell analysis maps the continuous regulatory landscape of human hematopoietic differentiation. Cell.

[bib26] Buenrostro J.D., Giresi P.G., Zaba L.C., Chang H.Y., Greenleaf W.J. (2013). Transposition of native chromatin for fast and sensitive epigenomic profiling of open chromatin, DNA-binding proteins and nucleosome position. Nat. Methods.

[bib27] Buenrostro J.D., Wu B., Chang H.Y., Greenleaf W.J. (2015). ATAC-seq: a method for assaying chromatin accessibility genome-wide. Curr. Protoc. Mol. Biol..

[bib28] Butler A., Hoffman P., Smibert P., Papalexi E., Satija R. (2018). Integrating single-cell transcriptomic data across different conditions, technologies, and species. Nat. Biotechnol..

[bib29] Butovsky O., Jedrychowski M.P., Moore C.S., Cialic R., Lanser A.J., Gabriely G., Koeglsperger T., Dake B., Wu P.M., Doykan C.E. (2014). Identification of a unique TGF-β-dependent molecular and functional signature in microglia. Nat. Neurosci..

[bib30] Cabezas-Wallscheid N., Klimmeck D., Hansson J., Lipka D.B., Reyes A., Wang Q., Weichenhan D., Lier A., von Paleske L., Renders S. (2014). Identification of regulatory networks in HSCs and their immediate progeny via integrated proteome, transcriptome, and DNA methylome analysis. Cell Stem Cell.

[bib31] Camacho D.M., Collins K.M., Powers R.K., Costello J.C., Collins J.J. (2018). Next-generation machine learning for biological networks. Cell.

[bib32] Cao M., Zhang H., Park J., Daniels N.M., Crovella M.E., Cowen L.J., Hescott B. (2013). Going the distance for protein function prediction: a new distance metric for protein interaction networks. PLoS One.

[bib33] Care M.A., Stephenson S.J., Barnes N.A., Fan I., Zougman A., El-Sherbiny Y.M., Vital E.M., Westhead D.R., Tooze R.M., Doody G.M. (2016). Network analysis identifies proinflammatory plasma cell polarization for secretion of ISG15 in human autoimmunity. J. Immunol..

[bib34] Chan T.E., Stumpf M.P.H., Babtie A.C. (2017). Gene regulatory network inference from single-cell data using multivariate Information measures. Cell Syst..

[bib35] Chapman N.M., Boothby M.R., Chi H. (2020). Metabolic coordination of T cell quiescence and activation. Nat. Rev. Immunol..

[bib36] Chaudhri V.K., Dienger-Stambaugh K., Wu Z., Shrestha M., Singh H. (2020). Charting the cis-regulome of activated B cells by coupling structural and functional genomics. Nat. Immunol..

[bib37] Chen K.H., Wang T.F., Hu Y.J. (2019). Protein-protein interaction prediction using a hybrid feature representation and a stacked generalization scheme. BMC Bioinformatics.

[bib38] Chen L., Flies D.B. (2013). Molecular mechanisms of T cell co-stimulation and co-inhibition. Nat. Rev. Immunol..

[bib267] Chen L., Kostadima M., Martens J.H.A., Canu G., Garcia S.P., Turro E., Downes K., Macaulay I.C., Bielczyk-Maczynska E., Coe S. (2014). Transcriptional diversity during lineage commitment of human blood progenitors. Science.

[bib39] Chen S., Mar J.C. (2018). Evaluating methods of inferring gene regulatory networks highlights their lack of performance for single cell gene expression data. BMC Bioinformatics.

[bib40] Cheng C., Easton J., Rosencrance C., Li Y., Ju B., Williams J., Mulder H.L., Pang Y., Chen W., Chen X. (2019). Latent cellular analysis robustly reveals subtle diversity in large-scale single-cell RNA-seq data. Nucleic Acids Res..

[bib41] Cho H., Berger B., Peng J. (2016). Compact integration of multi-network topology for functional analysis of genes. Cell Syst..

[bib254] Choi J., Baldwin T.M., Wong M., Bolden J.E., Fairfax K.A., Lucas E.C., Cole R., Biben C., Morgan C., Ramsay K.A. (2019). Haemopedia RNA-seq: a database of gene expression during haematopoiesis in mice and humans. Nucleic Acids Res..

[bib42] Choobdar S., Ahsen M.E., Crawford J., Tomasoni M., Fang T., Lamparter D., Lin J., Hescott B., Hu X., Mercer J. (2019). Assessment of network module identification across complex diseases. Nat. Methods.

[bib43] Chow A., Huggins M., Ahmed J., Hashimoto D., Lucas D., Kunisaki Y., Pinho S., Leboeuf M., Noizat C., van Rooijen N. (2013). CD169^+^ macrophages provide a niche promoting erythropoiesis under homeostasis and stress. Nat. Med..

[bib44] Clancy T., Hovig E. (2016). Profiling networks of distinct immune-cells in tumors. BMC Bioinformatics.

[bib45] Clark S.J., Lee H.J., Smallwood S.A., Kelsey G., Reik W. (2016). Single-cell epigenomics: powerful new methods for understanding gene regulation and cell identity. Genome Biol..

[bib46] Cocco M., Care M., Al-Maskari M., Doody G., Tooze R. (2019). A dichotomy in association of core transcription factors and gene regulation during the activated B-cell to plasmablast transition. bioRxiv.

[bib47] Cocco M., Stephenson S., Care M.A., Newton D., Barnes N.A., Davison A., Rawstron A., Westhead D.R., Doody G.M., Tooze R.M. (2012). In vitro generation of long-lived human plasma cells. J. Immunol..

[bib48] Corces M.R., Buenrostro J.D., Wu B., Greenside P.G., Chan S.M., Koenig J.L., Snyder M.P., Pritchard J.K., Kundaje A., Greenleaf W.J. (2016). Lineage-specific and single-cell chromatin accessibility charts human hematopoiesis and leukemia evolution. Nat. Genet..

[bib49] Costa F., Grün D., Backofen R. (2018). GraphDDP: a graph-embedding approach to detect differentiation pathways in single-cell-data using prior class knowledge. Nat. Commun..

[bib50] Cusanovich D.A., Hill A.J., Aghamirzaie D., Daza R.M., Pliner H.A., Berletch J.B., Filippova G.N., Huang X., Christiansen L., DeWitt W.S. (2018). A single-cell atlas of in vivo mammalian chromatin accessibility. Cell.

[bib51] Cyster J.G., Allen C.D.C. (2019). B cell responses: cell interaction dynamics and decisions. Cell.

[bib52] Datlinger P., Rendeiro A.F., Schmidl C., Krausgruber T., Traxler P., Klughammer J., Schuster L.C., Kuchler A., Alpar D., Bock C. (2017). Pooled CRISPR screening with single-cell transcriptome readout. Nat. Methods.

[bib253] de Graaf C.A., Choi J., Baldwin T.M., Bolden J.E., Fairfax K.A., Robinson A.J., Biben C., Morgan C., Ramsay K., Ng A.P. (2016). Haemopedia: an expression atlas of murine hematopoietic cells. Stem Cell Reports.

[bib53] DeTomaso D., Jones M.G., Subramaniam M., Ashuach T., Ye C.J., Yosef N. (2019). Functional interpretation of single cell similarity maps. Nat. Commun..

[bib54] Didier G., Brun C., Baudot A. (2015). Identifying communities from multiplex biological networks. PeerJ.

[bib55] Ding L., Morrison S.J. (2013). Haematopoietic stem cells and early lymphoid progenitors occupy distinct bone marrow niches. Nature.

[bib56] Ding L., Saunders T.L., Enikolopov G., Morrison S.J. (2012). Endothelial and perivascular cells maintain haematopoietic stem cells. Nature.

[bib57] DiSpirito J.R., Zemmour D., Ramanan D., Cho J., Zilionis R., Klein A.M., Benoist C., Mathis D. (2018). Molecular diversification of regulatory T cells in nonlymphoid tissues. Sci. Immunol..

[bib58] Dixit A., Parnas O., Li B., Chen J., Fulco C.P., Jerby-Arnon L., Marjanovic N.D., Dionne D., Burks T., Raychowdhury R. (2016). Perturb-seq: dissecting molecular circuits with scalable single-cell RNA profiling of pooled genetic screens. Cell.

[bib257] Drissen R., Buza-Vidas N., Woll P., Thongjuea S., Gambardella A., Giustacchini A., Mancini E., Zriwil A., Lutteropp M., Grover A. (2016). Distinct myeloid progenitor-differentiation pathways identified through single-cell RNA sequencing. Nat. Immunol..

[bib59] Du X., Wen J., Wang Y., Karmaus P.W.F., Khatamian A., Tan H., Li Y., Guy C., Nguyen T.M., Dhungana Y. (2018). Hippo/Mst signalling couples metabolic state and immune function of CD8α^+^ dendritic cells. Nature.

[bib60] Dudziak D., Kamphorst A.O., Heidkamp G.F., Buchholz V.R., Trumpfheller C., Yamazaki S., Cheong C., Liu K., Lee H.W., Park C.G. (2007). Differential antigen processing by dendritic cell subsets in vivo. Science.

[bib61] Duren Z., Chen X., Jiang R., Wang Y., Wong W.H. (2017). Modeling gene regulation from paired expression and chromatin accessibility data. Proc. Natl. Acad. Sci. U S A.

[bib62] Efremova M., Teichmann S.A. (2020). Computational methods for single-cell omics across modalities. Nat. Methods.

[bib63] Eisenbarth S.C. (2019). Dendritic cell subsets in T cell programming: location dictates function. Nat. Rev. Immunol..

[bib64] Eng C.L., Lawson M., Zhu Q., Dries R., Koulena N., Takei Y., Yun J., Cronin C., Karp C., Yuan G.C. (2019). Transcriptome-scale super-resolved imaging in tissues by RNA seqFISH. Nature.

[bib65] Eng C.L., Shah S., Thomassie J., Cai L. (2017). Profiling the transcriptome with RNA SPOTs. Nat. Methods.

[bib66] Epelman S., Lavine K.J., Randolph G.J. (2014). Origin and functions of tissue macrophages. Immunity.

[bib67] Franzosa E., Linghu B., Xia Y. (2009). Computational reconstruction of protein-protein interaction networks: algorithms and issues. Methods Mol. Biol..

[bib68] Furey T.S. (2012). ChIP-seq and beyond: new and improved methodologies to detect and characterize protein–DNA interactions. Nat. Rev. Genet..

[bib69] Gabryšová L., Alvarez-Martinez M., Luisier R., Cox L.S., Sodenkamp J., Hosking C., Pérez-Mazliah D., Whicher C., Kannan Y., Potempa K. (2018). c-Maf controls immune responses by regulating disease-specific gene networks and repressing IL-2 in CD4^+^ T cells. Nat. Immunol..

[bib70] Gajewski T.F., Schreiber H., Fu Y.X. (2013). Innate and adaptive immune cells in the tumor microenvironment. Nat. Immunol..

[bib71] Gaublomme J.T., Yosef N., Lee Y., Gertner R.S., Yang L.V., Wu C., Pandolfi P.P., Mak T., Satija R., Shalek A.K. (2015). Single-cell genomics unveils critical regulators of Th17 cell pathogenicity. Cell.

[bib72] Gaulton K.J., Nammo T., Pasquali L., Simon J.M., Giresi P.G., Fogarty M.P., Panhuis T.M., Mieczkowski P., Secchi A., Bosco D. (2010). A map of open chromatin in human pancreatic islets. Nat. Genet..

[bib73] Geiger R., Rieckmann J.C., Wolf T., Basso C., Feng Y., Fuhrer T., Kogadeeva M., Picotti P., Meissner F., Mann M. (2016). L-Arginine modulates T cell metabolism and enhances survival and anti-tumor activity. Cell.

[bib74] Gerstein M.B., Kundaje A., Hariharan M., Landt S.G., Yan K.K., Cheng C., Mu X.J., Khurana E., Rozowsky J., Alexander R. (2012). Architecture of the human regulatory network derived from ENCODE data. Nature.

[bib268] Giladi A., Amit I. (2018). Single-cell genomics: a stepping stone for future immunology discoveries. Cell.

[bib75] Giladi A., Cohen M., Medaglia C., Baran Y., Li B., Zada M., Bost P., Blecher-Gonen R., Salame T.M., Mayer J.U. (2020). Dissecting cellular crosstalk by sequencing physically interacting cells. Nat. Biotechnol..

[bib76] Giladi A., Paul F., Herzog Y., Lubling Y., Weiner A., Yofe I., Jaitin D., Cabezas-Wallscheid N., Dress R., Ginhoux F. (2018). Single-cell characterization of haematopoietic progenitors and their trajectories in homeostasis and perturbed haematopoiesis. Nat. Cell Biol..

[bib77] Gilbert L.A., Horlbeck M.A., Adamson B., Villalta J.E., Chen Y., Whitehead E.H., Guimaraes C., Panning B., Ploegh H.L., Bassik M.C. (2014). Genome-scale CRISPR-mediated control of gene repression and activation. Cell.

[bib78] Ginhoux F., Greter M., Leboeuf M., Nandi S., See P., Gokhan S., Mehler M.F., Conway S.J., Ng L.G., Stanley E.R. (2010). Fate mapping analysis reveals that adult microglia derive from primitive macrophages. Science.

[bib79] Gomez Perdiguero E., Klapproth K., Schulz C., Busch K., Azzoni E., Crozet L., Garner H., Trouillet C., de Bruijn M.F., Geissmann F. (2015). Tissue-resident macrophages originate from yolk-sac-derived erythro-myeloid progenitors. Nature.

[bib80] Gosselin D., Link V.M., Romanoski C.E., Fonseca G.J., Eichenfield D.Z., Spann N.J., Stender J.D., Chun H.B., Garner H., Geissmann F. (2014). Environment drives selection and function of enhancers controlling tissue-specific macrophage identities. Cell.

[bib81] Grandclaudon M., Perrot-Dockès M., Trichot C., Karpf L., Abouzid O., Chauvin C., Sirven P., Abou-Jaoudé W., Berger F., Hupé P. (2019). A qauantitative multivariate model of human dendritic cell-T helper cell communication. Cell.

[bib82] Greenbaum A., Hsu Y.M., Day R.B., Schuettpelz L.G., Christopher M.J., Borgerding J.N., Nagasawa T., Link D.C. (2013). CXCL12 in early mesenchymal progenitors is required for haematopoietic stem-cell maintenance. Nature.

[bib83] Gu S., Johnson J., Faisal F.E., Milenković T. (2018). From homogeneous to heterogeneous network alignment via colored graphlets. Sci. Rep..

[bib84] Gut G., Herrmann M.D., Pelkmans L. (2018). Multiplexed protein maps link subcellular organization to cellular states. Science.

[bib85] Guzzi P.H., Milenkovic T. (2018). Survey of local and global biological network alignment: the need to reconcile the two sides of the same coin. Brief. Bioinform..

[bib86] Haldar M., Kohyama M., So A.Y., Kc W., Wu X., Briseño C.G., Satpathy A.T., Kretzer N.M., Arase H., Rajasekaran N.S. (2014). Heme-mediated SPI-C induction promotes monocyte differentiation into iron-recycling macrophages. Cell.

[bib87] Han X., Wang R., Zhou Y., Fei L., Sun H., Lai S., Saadatpour A., Zhou Z., Chen H., Ye F. (2018). Mapping the mouse cell atlas by microwell-seq. Cell.

[bib88] Hartmann F.J., Babdor J., Gherardini P.F., Amir E.D., Jones K., Sahaf B., Marquez D.M., Krutzik P., O'Donnell E., Sigal N. (2019). Comprehensive immune monitoring of clinical trials to advance human immunotherapy. Cell Rep..

[bib89] Hartwell L.H., Hopfield J.J., Leibler S., Murray A.W. (1999). From molecular to modular cell biology. Nature.

[bib90] Hashimoto D., Chow A., Noizat C., Teo P., Beasley M.B., Leboeuf M., Becker C.D., See P., Price J., Lucas D. (2013). Tissue-resident macrophages self-maintain locally throughout adult life with minimal contribution from circulating monocytes. Immunity.

[bib91] Hein M.Y., Hubner N.C., Poser I., Cox J., Nagaraj N., Toyoda Y., Gak I.A., Weisswange I., Mansfeld J., Buchholz F. (2015). A human interactome in three quantitative dimensions organized by stoichiometries and abundances. Cell.

[bib92] Helmink B.A., Reddy S.M., Gao J., Zhang S., Basar R., Thakur R., Yizhak K., Sade-Feldman M., Blando J., Han G. (2020). B cells and tertiary lymphoid structures promote immunotherapy response. Nature.

[bib93] Henriksson J., Chen X., Gomes T., Ullah U., Meyer K.B., Miragaia R., Duddy G., Pramanik J., Yusa K., Lahesmaa R. (2019). Genome-wide CRISPR screens in T helper cells reveal pervasive crosstalk between activation and differentiation. Cell.

[bib94] Hernández-Santos N., Gaffen S.L. (2012). Th17 cells in immunity to Candida albicans. Cell Host Microbe.

[bib95] Hirota K., Duarte J.H., Veldhoen M., Hornsby E., Li Y., Cua D.J., Ahlfors H., Wilhelm C., Tolaini M., Menzel U. (2011). Fate mapping of IL-17-producing T cells in inflammatory responses. Nat. Immunol..

[bib96] Hirota K., Turner J.E., Villa M., Duarte J.H., Demengeot J., Steinmetz O.M., Stockinger B. (2013). Plasticity of T_H_17 cells in Peyer's patches is responsible for the induction of T cell-dependent IgA responses. Nat. Immunol..

[bib97] Hoeffel G., Chen J., Lavin Y., Low D., Almeida F.F., See P., Beaudin A.E., Lum J., Low I., Forsberg E.C. (2015). C-Myb^+^ erythro-myeloid progenitor-derived fetal monocytes give rise to adult tissue-resident macrophages. Immunity.

[bib98] Hoggatt J., Kfoury Y., Scadden D.T. (2016). Hematopoietic stem cell niche in health and disease. Annu. Rev. Pathol..

[bib263] Huang P., Keller C.A., Giardine B., Grevet J.D., Davies J.O.J., Hughes J.R., Kurita R., Nakamura Y., Hardison R.C., Blobel G.A. (2017). Comparative analysis of three-dimensional chromosomal architecture identifies a novel fetal hemoglobin regulatory element. Genes Dev..

[bib99] Huttlin E.L., Bruckner R.J., Paulo J.A., Cannon J.R., Ting L., Baltier K., Colby G., Gebreab F., Gygi M.P., Parzen H. (2017). Architecture of the human interactome defines protein communities and disease networks. Nature.

[bib100] Iacono G., Massoni-Badosa R., Heyn H. (2019). Single-cell transcriptomics unveils gene regulatory network plasticity. Genome Biol..

[bib101] Iwasaki H., Akashi K. (2007). Myeloid lineage commitment from the hematopoietic stem cell. Immunity.

[bib102] Jaitin D.A., Weiner A., Yofe I., Lara-Astiaso D., Keren-Shaul H., David E., Salame T.M., Tanay A., van Oudenaarden A., Amit I. (2016). Dissecting immune circuits by linking CRISPR-pooled screens with single-cell RNA-seq. Cell.

[bib103] Jansen C., Ramirez R.N., El-Ali N.C., Gomez-Cabrero D., Tegner J., Merkenschlager M., Conesa A., Mortazavi A. (2019). Building gene regulatory networks from scATAC-seq and scRNA-seq using Linked Self Organizing Maps. PLoS Comput. Biol..

[bib104] Jansen R., Greenbaum D., Gerstein M. (2002). Relating whole-genome expression data with protein-protein interactions. Genome Res..

[bib105] Jego G., Palucka A.K., Blanck J.P., Chalouni C., Pascual V., Banchereau J. (2003). Plasmacytoid dendritic cells induce plasma cell differentiation through type I interferon and interleukin 6. Immunity.

[bib106] Ji H., Ehrlich L.I., Seita J., Murakami P., Doi A., Lindau P., Lee H., Aryee M.J., Irizarry R.A., Kim K. (2010). Comprehensive methylome map of lineage commitment from haematopoietic progenitors. Nature.

[bib107] Karmaus P.W.F., Chen X., Lim S.A., Herrada A.A., Nguyen T.M., Xu B., Dhungana Y., Rankin S., Chen W., Rosencrance C. (2019). Metabolic heterogeneity underlies reciprocal fates of TH17 cell stemness and plasticity. Nature.

[bib108] Kc K., Li R., Cui F., Yu Q., Haake A.R. (2019). GNE: a deep learning framework for gene network inference by aggregating biological information. BMC Syst. Biol..

[bib109] Khatamian A., Paull E.O., Califano A., Yu J. (2019). SJARACNe: a scalable software tool for gene network reverse engineering from big data. Bioinformatics.

[bib110] Kholodenko B., Yaffe M.B., Kolch W. (2012). Computational approaches for analyzing information flow in biological networks. Sci. Signal..

[bib111] Kim S.I., Bresnick E.H. (2007). Transcriptional control of erythropoiesis: emerging mechanisms and principles. Oncogene.

[bib112] Kirouac D.C., Ito C., Csaszar E., Roch A., Yu M., Sykes E.A., Bader G.D., Zandstra P.W. (2010). Dynamic interaction networks in a hierarchically organized tissue. Mol. Syst. Biol..

[bib113] Kiselev V.Y., Andrews T.S., Hemberg M. (2019). Challenges in unsupervised clustering of single-cell RNA-seq data. Nat. Rev. Genet..

[bib114] Kiselev V.Y., Kirschner K., Schaub M.T., Andrews T., Yiu A., Chandra T., Natarajan K.N., Reik W., Barahona M., Green A.R. (2017). SC3: consensus clustering of single-cell RNA-seq data. Nat. Methods.

[bib115] Kohyama M., Ise W., Edelson B.T., Wilker P.R., Hildner K., Mejia C., Frazier W.A., Murphy T.L., Murphy K.M. (2009). Role for Spi-C in the development of red pulp macrophages and splenic iron homeostasis. Nature.

[bib116] Kondratova M., Czerwinska U., Sompairac N., Amigorena S.D., Soumelis V., Barillot E., Zinovyev A., Kuperstein I. (2019). A multiscale signalling network map of innate immune response in cancer reveals cell heterogeneity signatures. Nat. Commun..

[bib117] Korn T., Bettelli E., Oukka M., Kuchroo V.K. (2009). IL-17 and Th17 cells. Annu. Rev. Immunol..

[bib118] Krishnaswamy S., Spitzer M.H., Mingueneau M., Bendall S.C., Litvin O., Stone E., Pe'er D., Nolan G.P. (2014). Conditional density-based analysis of T cell signaling in single-cell data. Science.

[bib119] Kumar M.P., Du J., Lagoudas G., Jiao Y., Sawyer A., Drummond D.C., Lauffenburger D.A., Raue A. (2018). Analysis of single-cell RNA-seq identifies cell-cell communication associated with tumor characteristics. Cell Rep..

[bib120] Kunisaki Y., Bruns I., Scheiermann C., Ahmed J., Pinho S., Zhang D., Mizoguchi T., Wei Q., Lucas D., Ito K. (2013). Arteriolar niches maintain haematopoietic stem cell quiescence. Nature.

[bib121] La Manno G., Soldatov R., Zeisel A., Braun E., Hochgerner H., Petukhov V., Lidschreiber K., Kastriti M.E., Lönnerberg P., Furlan A. (2018). RNA velocity of single cells. Nature.

[bib122] Lai Z., Tsugawa H., Wohlgemuth G., Mehta S., Mueller M., Zheng Y., Ogiwara A., Meissen J., Showalter M., Takeuchi K. (2018). Identifying metabolites by integrating metabolome databases with mass spectrometry cheminformatics. Nat. Methods.

[bib264] Lalonde S., Stone O.A., Lessard S., Lavertu A., Desjardins J., Beaudoin M., Rivas M., Stainier D.Y.R., Lettre G. (2017). Frameshift indels introduced by genome editing can lead to in-frame exon skipping. PLoS One.

[bib123] Langfelder P., Horvath S. (2008). WGCNA: an R package for weighted correlation network analysis. BMC Bioinformatics.

[bib124] Lara-Astiaso D., Weiner A., Lorenzo-Vivas E., Zaretsky I., Jaitin D.A., David E., Keren-Shaul H., Mildner A., Winter D., Jung S. (2014). Chromatin state dynamics during blood formation. Science.

[bib125] Laurenti E., Doulatov S., Zandi S., Plumb I., Chen J., April C., Fan J.B., Dick J.E. (2013). The transcriptional architecture of early human hematopoiesis identifies multilevel control of lymphoid commitment. Nat. Immunol..

[bib126] Laurenti E., Göttgens B. (2018). From haematopoietic stem cells to complex differentiation landscapes. Nature.

[bib127] Lavin Y., Winter D., Blecher-Gonen R., David E., Keren-Shaul H., Merad M., Jung S., Amit I. (2014). Tissue-resident macrophage enhancer landscapes are shaped by the local microenvironment. Cell.

[bib128] Lee Y., Awasthi A., Yosef N., Quintana F.J., Xiao S., Peters A., Wu C., Kleinewietfeld M., Kunder S., Hafler D.A. (2012). Induction and molecular signature of pathogenic TH17 cells. Nat. Immunol..

[bib129] Lee Y.K., Turner H., Maynard C.L., Oliver J.R., Chen D.Q., Elson C.O., Weaver C.T. (2009). Late developmental plasticity in the T helper 17 lineage. Immunity.

[bib130] Levine J.H., Simonds E.F., Bendall S.C., Davis K.L., Amir el A.D., Tadmor M.D., Litvin O., Fienberg H.G., Jager A., Zunder E.R. (2015). Data-driven phenotypic dissection of AML reveals progenitor-like cells that correlate with prognosis. Cell.

[bib131] Li D., Gao J. (2019). Towards perturbation prediction of biological networks using deep learning. Sci. Rep..

[bib132] Li Q., Barres B.A. (2018). Microglia and macrophages in brain homeostasis and disease. Nat. Rev. Immunol..

[bib133] Liu L., Leng L., Liu C., Lu C., Yuan Y., Wu L., Gong F., Zhang S., Wei X., Wang M. (2019). An integrated chromatin accessibility and transcriptome landscape of human pre-implantation embryos. Nat. Commun..

[bib134] Liu L., Liu C., Quintero A., Wu L., Yuan Y., Wang M., Cheng M., Leng L., Xu L., Dong G. (2019). Deconvolution of single-cell multi-omics layers reveals regulatory heterogeneity. Nat. Commun..

[bib135] Liu Y., Beyer A., Aebersold R. (2016). On the dependency of cellular protein levels on mRNA abundance. Cell.

[bib265] Liu X., Zhang Y., Ni M., Cao H., Signer R.A.J., Li D., Li M., Gu Z., Hu Z., Dickerson K.E. (2017). Regulation of mitochondrial biogenesis in erythropoiesis by mTORC1-mediated protein translation. Nat. Cell Biol..

[bib261] Ludwig L.S., Lareau C.A., Bao E.L., Nandakumar S.K., Muus C., Ulirsch J.C., Chowdhary K., Buenrostro J.D., Mohandas N., An X. (2019). Transcriptional states and chromatin accessibility underlying human erythropoiesis. Cell Rep..

[bib136] Luecken M.D., Theis F.J. (2019). Current best practices in single-cell RNA-seq analysis: a tutorial. Mol. Syst. Biol..

[bib137] Mann M., Kulak N.A., Nagaraj N., Cox J. (2013). The coming age of complete, accurate, and ubiquitous proteomes. Mol. Cell.

[bib138] Margolin A.A., Nemenman I., Basso K., Wiggins C., Stolovitzky G., Dalla Favera R., Califano A. (2006). ARACNE: an algorithm for the reconstruction of gene regulatory networks in a mammalian cellular context. BMC Bioinformatics.

[bib139] Markiewski M.M., DeAngelis R.A., Benencia F., Ricklin-Lichtsteiner S.K., Koutoulaki A., Gerard C., Coukos G., Lambris J.D. (2008). Modulation of the antitumor immune response by complement. Nat. Immunol..

[bib140] Matsumoto H., Kiryu H., Furusawa C., Ko M.S.H., Ko S.B.H., Gouda N., Hayashi T., Nikaido I. (2017). SCODE: an efficient regulatory network inference algorithm from single-cell RNA-Seq during differentiation. Bioinformatics.

[bib141] Mellacheruvu D., Wright Z., Couzens A.L., Lambert J.P., St-Denis N.A., Li T., Miteva Y.V., Hauri S., Sardiu M.E., Low T.Y. (2013). The CRAPome: a contaminant repository for affinity purification-mass spectrometry data. Nat. Methods.

[bib142] Méndez-Ferrer S., Michurina T.V., Ferraro F., Mazloom A.R., Macarthur B.D., Lira S.A., Scadden D.T., Ma'ayan A., Enikolopov G.N., Frenette P.S. (2010). Mesenchymal and haematopoietic stem cells form a unique bone marrow niche. Nature.

[bib143] Mendez A., Mendoza L. (2016). A network model to describe the terminal differentiation of B cells. PLoS Comput. Biol..

[bib144] Mezger A., Klemm S., Mann I., Brower K., Mir A., Bostick M., Farmer A., Fordyce P., Linnarsson S., Greenleaf W. (2018). High-throughput chromatin accessibility profiling at single-cell resolution. Nat. Commun..

[bib145] Mifsud B., Tavares-Cadete F., Young A.N., Sugar R., Schoenfelder S., Ferreira L., Wingett S.W., Andrews S., Grey W., Ewels P.A. (2015). Mapping long-range promoter contacts in human cells with high-resolution capture Hi-C. Nat. Genet..

[bib146] Mignone P., Pio G., D'Elia D., Ceci M. (2019). Exploiting transfer learning for the reconstruction of the human gene regulatory network. Bioinformatics.

[bib147] Miraldi E.R., Pokrovskii M., Watters A., Castro D.M., De Veaux N., Hall J.A., Lee J.Y., Ciofani M., Madar A., Carriero N. (2019). Leveraging chromatin accessibility for transcriptional regulatory network inference in T helper 17 cells. Genome Res..

[bib148] Mochida K., Koda S., Inoue K., Nishii R. (2018). Statistical and machine learning approaches to predict gene regulatory networks from transcriptome datasets. Front. Plant Sci..

[bib149] Moffitt J.R., Bambah-Mukku D., Eichhorn S.W., Vaughn E., Shekhar K., Perez J.D., Rubinstein N.D., Hao J., Regev A., Dulac C. (2018). Molecular, spatial, and functional single-cell profiling of the hypothalamic preoptic region. Science.

[bib150] Moncada R., Barkley D., Wagner F., Chiodin M., Devlin J.C., Baron M., Hajdu C.H., Simeone D.M., Yanai I. (2020). Integrating microarray-based spatial transcriptomics and single-cell RNA-seq reveals tissue architecture in pancreatic ductal adenocarcinomas. Nat. Biotechnol..

[bib151] Morgens D.W., Deans R.M., Li A., Bassik M.C. (2016). Systematic comparison of CRISPR/Cas9 and RNAi screens for essential genes. Nat. Biotechnol..

[bib152] Morris S.A., Cahan P., Li H., Zhao A.M., San Roman A.K., Shivdasani R.A., Collins J.J., Daley G.Q. (2014). Dissecting engineered cell types and enhancing cell fate conversion via CellNet. Cell.

[bib153] Morrison S.J., Scadden D.T. (2014). The bone marrow niche for haematopoietic stem cells. Nature.

[bib154] Mumbach M.R., Rubin A.J., Flynn R.A., Dai C., Khavari P.A., Greenleaf W.J., Chang H.Y. (2016). HiChIP: efficient and sensitive analysis of protein-directed genome architecture. Nat. Methods.

[bib155] Nassar, H., and Gleich, D.F. (2017). Multimodal network alignment. In Proceedings of the 2017 SIAM International Conference on Data Mining, Houston, TX, April 2017, pp 615–623.

[bib156] Nelson W., Zitnik M., Wang B., Leskovec J., Goldenberg A., Sharan R. (2019). To embed or not: network embedding as a paradigm in computational biology. Front. Genet..

[bib157] Neph S., Stergachis A.B., Reynolds A., Sandstrom R., Borenstein E., Stamatoyannopoulos J.A. (2012). Circuitry and dynamics of human transcription factor regulatory networks. Cell.

[bib256] Nestorowa S., Hamey F.K., Pijuan Sala B., Diamanti E., Shepherd M., Laurenti E., Wilson N.K., Kent D.G., Gottgens B. (2016). A single-cell resolution map of mouse hematopoietic stem and progenitor cell differentiation. Blood.

[bib158] Nitzan M., Karaiskos N., Friedman N., Rajewsky N. (2019). Gene expression cartography. Nature.

[bib159] Northrup D.L., Zhao K. (2011). Application of ChIP-Seq and related techniques to the study of immune function. Immunity.

[bib160] Notta F., Zandi S., Takayama N., Dobson S., Gan O.I., Wilson G., Kaufmann K.B., McLeod J., Laurenti E., Dunant C.F. (2016). Distinct routes of lineage development reshape the human blood hierarchy across ontogeny. Science.

[bib161] Novershtern N., Subramanian A., Lawton L.N., Mak R.H., Haining W.N., McConkey M.E., Habib N., Yosef N., Chang C.Y., Shay T. (2011). Densely interconnected transcriptional circuits control cell states in human hematopoiesis. Cell.

[bib162] Nutt S.L., Hodgkin P.D., Tarlinton D.M., Corcoran L.M. (2015). The generation of antibody-secreting plasma cells. Nat. Rev. Immunol..

[bib163] Okabe Y., Medzhitov R. (2014). Tissue-specific signals control reversible program of localization and functional polarization of macrophages. Cell.

[bib260] Olsson A., Venkatasubramanian M., Chaudhri V.K., Aronow B.J., Salomonis N., Singh H., Grimes H.L. (2016). Single-cell analysis of mixed-lineage states leading to a binary cell fate choice. Nature.

[bib164] Orkin S.H., Zon L.I. (2008). Hematopoiesis: an evolving paradigm for stem cell biology. Cell.

[bib165] Paolicelli R.C., Bolasco G., Pagani F., Maggi L., Scianni M., Panzanelli P., Giustetto M., Ferreira T.A., Guiducci E., Dumas L. (2011). Synaptic pruning by microglia is necessary for normal brain development. Science.

[bib166] Park J.E., Botting R.A., Dominguez Conde C., Popescu D.M., Lavaert M., Kunz D.J., Goh I., Stephenson E., Ragazzini R., Tuck E. (2020). A cell atlas of human thymic development defines T cell repertoire formation. Science.

[bib167] Park P.J. (2009). ChIP-seq: advantages and challenges of a maturing technology. Nat. Rev. Genet..

[bib168] Paul F., Arkin Y., Giladi A., Jaitin D.A., Kenigsberg E., Keren-Shaul H., Winter D., Lara-Astiaso D., Gury M., Weiner A. (2015). Transcriptional heterogeneity and lineage commitment in myeloid progenitors. Cell.

[bib169] Pons P., Latapy M. (2005). Computing communities in large networks using random walks. Lecture Notes Comput. Sci..

[bib170] Raghavan U.N., Albert R., Kumara S. (2007). Near linear time algorithm to detect community structures in large-scale networks. Phys. Rev. E Stat. Nonlin. Soft Matter Phys..

[bib171] Ramilowski J.A., Goldberg T., Harshbarger J., Kloppmann E., Lizio M., Satagopam V.P., Itoh M., Kawaji H., Carninci P., Rost B. (2015). A draft network of ligand-receptor-mediated multicellular signalling in human. Nat. Commun..

[bib172] Ramirez R.N., El-Ali N.C., Mager M.A., Wyman D., Conesa A., Mortazavi A. (2017). Dynamic gene regulatory networks of human myeloid differentiation. Cell Syst..

[bib173] Regev A., Teichmann S.A., Lander E.S., Amit I., Benoist C., Birney E., Bodenmiller B., Campbell P., Carninci P., Clatworthy M. (2017). The human cell atlas. eLife.

[bib174] Rendeiro A.F., Schmidl C., Strefford J.C., Walewska R., Davis Z., Farlik M., Oscier D., Bock C. (2016). Chromatin accessibility maps of chronic lymphocytic leukaemia identify subtype-specific epigenome signatures and transcription regulatory networks. Nat. Commun..

[bib175] Rieckmann J.C., Geiger R., Hornburg D., Wolf T., Kveler K., Jarrossay D., Sallusto F., Shen-Orr S.S., Lanzavecchia A., Mann M. (2017). Social network architecture of human immune cells unveiled by quantitative proteomics. Nat. Immunol..

[bib176] Kundaje A., Meuleman W., Ernst J., Bilenky M., Yen A., Heravi-Moussavi A., Kheradpour P., Zhang Z., Wang J., Roadmap Epigenomics Consortium (2015). Integrative analysis of 111 reference human epigenomes. Nature.

[bib177] Rodriques S.G., Stickels R.R., Goeva A., Martin C.A., Murray E., Vanderburg C.R., Welch J., Chen L.M., Chen F., Macosko E.Z. (2019). Slide-seq: a scalable technology for measuring genome-wide expression at high spatial resolution. Science.

[bib178] Rosvall M., Bergstrom C.T. (2011). Multilevel compression of random walks on networks reveals hierarchical organization in large integrated systems. PLoS One.

[bib179] Rotem A., Ram O., Shoresh N., Sperling R.A., Goren A., Weitz D.A., Bernstein B.E. (2015). Single-cell ChIP-seq reveals cell subpopulations defined by chromatin state. Nat. Biotechnol..

[bib180] Rothenberg E.V. (2014). Transcriptional control of early T and B cell developmental choices. Annu. Rev. Immunol..

[bib181] Roux K.J., Kim D.I., Burke B. (2013). BioID: a screen for protein-protein interactions. Curr. Protoc. Protein Sci..

[bib182] Saelens W., Cannoodt R., Todorov H., Saeys Y. (2019). A comparison of single-cell trajectory inference methods. Nat. Biotechnol..

[bib183] Saez-Rodriguez J., MacNamara A., Cook S. (2015). Modeling signaling networks to advance new cancer therapies. Annu. Rev. Biomed. Eng..

[bib184] Sanjuan-Pla A., Macaulay I.C., Jensen C.T., Woll P.S., Luis T.C., Mead A., Moore S., Carella C., Matsuoka S., Bouriez Jones T. (2013). Platelet-biased stem cells reside at the apex of the haematopoietic stem-cell hierarchy. Nature.

[bib185] Saraph V., Milenković T. (2014). MAGNA: maximizing accuracy in global network alignment. Bioinformatics.

[bib186] Saravia J., Chapman N.M., Chi H. (2019). Helper T cell differentiation. Cell Mol Immunol..

[bib187] Sauteraud R., Dashevskiy L., Finak G., Gottardo R. (2016). ImmuneSpace: enabling integrative modeling of human immunological data. J. Immunol..

[bib188] Scharer C.D., Barwick B.G., Guo M., Bally A.P.R., Boss J.M. (2018). Plasma cell differentiation is controlled by multiple cell division-coupled epigenetic programs. Nat. Commun..

[bib258] Schlitzer A., Sivakamasundari V., Chen J., Sumatoh H.R., Schreuder J., Lum J., Malleret B., Zhang S., Larbi A., Zolezzi F. (2015). Identification of cDC1- and cDC2-committed DC progenitors reveals early lineage priming at the common DC progenitor stage in the bone marrow. Nat. Immunol..

[bib189] Schoenfelder S., Fraser P. (2019). Long-range enhancer-promoter contacts in gene expression control. Nat. Rev. Genet..

[bib190] Schulz C., Gomez Perdiguero E., Chorro L., Szabo-Rogers H., Cagnard N., Kierdorf K., Prinz M., Wu B., Jacobsen S.E., Pollard J.W. (2012). A lineage of myeloid cells independent of Myb and hematopoietic stem cells. Science.

[bib191] Shalem O., Sanjana N.E., Zhang F. (2015). High-throughput functional genomics using CRISPR-Cas9. Nat. Rev. Genet..

[bib192] Shay T., Kang J. (2013). Immunological genome Project and systems immunology. Trends Immunol..

[bib193] Sheikh B.N., Bondareva O., Guhathakurta S., Tsang T.H., Sikora K., Aizarani N., Sagar H., Grün D., Hein L. (2019). Systematic identification of cell-cell communication networks in the developing brain. iScience.

[bib194] Sheng J., Ruedl C., Karjalainen K. (2015). Most tissue-resident macrophages except microglia are derived from fetal hematopoietic stem cells. Immunity.

[bib195] Shi H., Chapman N.M., Wen J., Guy C., Long L., Dhungana Y., Rankin S., Pelletier S., Vogel P., Wang H. (2019). Amino acids license kinase mTORC1 activity and Treg cell function via small G proteins Rag and Rheb. Immunity.

[bib196] Shi H., Chi H. (2019). Metabolic control of Treg cell stability, plasticity, and tissue-specific heterogeneity. Front. Immunol..

[bib197] Shi H., Liu C., Tan H., Li Y., Nguyen T.M., Dhungana Y., Guy C., Vogel P., Neale G., Rankin S. (2018). Hippo kinases Mst1 and Mst2 sense and amplify IL-2R-STAT5 signaling in regulatory T cells to establish stable regulatory activity. Immunity.

[bib198] Shi W., Liao Y., Willis S.N., Taubenheim N., Inouye M., Tarlinton D.M., Smyth G.K., Hodgkin P.D., Nutt S.L., Corcoran L.M. (2015). Transcriptional profiling of mouse B cell terminal differentiation defines a signature for antibody-secreting plasma cells. Nat. Immunol..

[bib199] Singh H., Khan A.A., Dinner A.R. (2014). Gene regulatory networks in the immune system. Trends Immunol..

[bib200] Singh R., Xu J., Berger B. (2008). Global alignment of multiple protein interaction networks with application to functional orthology detection. Proc. Natl. Acad. Sci. U S A.

[bib201] Smillie C.S., Biton M., Ordovas-Montanes J., Sullivan K.M., Burgin G., Graham D.B., Herbst R.H., Rogel N., Slyper M., Waldman J. (2019). Intra- and inter-cellular rewiring of the human colon during ulcerative colitis. Cell.

[bib202] Soucie E.L., Weng Z., Geirsdóttir L., Molawi K., Maurizio J., Fenouil R., Mossadegh-Keller N., Gimenez G., VanHille L., Beniazza M. (2016). Lineage-specific enhancers activate self-renewal genes in macrophages and embryonic stem cells. Science.

[bib203] Spitzer M.H., Nolan G.P. (2016). Mass cytometry: single cells, many features. Cell.

[bib204] Stahl P.L., Salmen F., Vickovic S., Lundmark A., Navarro J.F., Magnusson J., Giacomello S., Asp M., Westholm J.O., Huss M. (2016). Visualization and analysis of gene expression in tissue sections by spatial transcriptomics. Science.

[bib205] Stark C., Breitkreutz B.J., Reguly T., Boucher L., Breitkreutz A., Tyers M. (2006). BioGRID: a general repository for interaction datasets. Nucleic Acids Res..

[bib206] Stegle O., Teichmann S.A., Marioni J.C. (2015). Computational and analytical challenges in single-cell transcriptomics. Nat. Rev. Genet..

[bib207] Stevens T.J., Lando D., Basu S., Atkinson L.P., Cao Y., Lee S.F., Leeb M., Wohlfahrt K.J., Boucher W., O'Shaughnessy-Kirwan A. (2017). 3D structures of individual mammalian genomes studied by single-cell Hi-C. Nature.

[bib208] Stewart B.J., Ferdinand J.R., Young M.D., Mitchell T.J., Loudon K.W., Riding A.M., Richoz N., Frazer G.L., Staniforth J.U.L., Vieira Braga F.A. (2019). Spatiotemporal immune zonation of the human kidney. Science.

[bib209] Stewart E., McEvoy J., Wang H., Chen X., Honnell V., Ocarz M., Gordon B., Dapper J., Blankenship K., Yang Y. (2018). Identification of therapeutic targets in rhabdomyosarcoma through integrated genomic, epigenomic, and proteomic analyses. Cancer Cell.

[bib210] Stoeckius M., Hafemeister C., Stephenson W., Houck-Loomis B., Chattopadhyay P.K., Swerdlow H., Satija R., Smibert P. (2017). Simultaneous epitope and transcriptome measurement in single cells. Nat. Methods.

[bib211] Stritesky G.L., Jameson S.C., Hogquist K.A. (2012). Selection of self-reactive T cells in the thymus. Annu. Rev. Immunol..

[bib269] Stubbington M.J.T., Rozenblatt-Rosen O., Regev A., Teichmann S.A. (2017). Single-cell transcriptomics to explore the immune system in health and disease. Science.

[bib212] Subelj L., Bajec M. (2011). Unfolding communities in large complex networks: combining defensive and offensive label propagation for core extraction. Phys. Rev. E Stat. Nonlin. Soft Matter Phys..

[bib213] Sugiyama T., Kohara H., Noda M., Nagasawa T. (2006). Maintenance of the hematopoietic stem cell pool by CXCL12-CXCR4 chemokine signaling in bone marrow stromal cell niches. Immunity.

[bib214] Szklarczyk D., Franceschini A., Wyder S., Forslund K., Heller D., Huerta-Cepas J., Simonovic M., Roth A., Santos A., Tsafou K.P. (2015). STRING v10: protein-protein interaction networks, integrated over the tree of life. Nucleic Acids Res..

[bib215] Szklarczyk D., Gable A.L., Lyon D., Junge A., Wyder S., Huerta-Cepas J., Simonovic M., Doncheva N.T., Morris J.H., Bork P. (2019). STRING v11: protein-protein association networks with increased coverage, supporting functional discovery in genome-wide experimental datasets. Nucleic Acids Res..

[bib216] Tan H., Yang K., Li Y., Shaw T.I., Wang Y., Blanco D.B., Wang X., Cho J.H., Wang H., Rankin S. (2017). Integrative proteomics and phosphoproteomics profiling reveals dynamic signaling networks and bioenergetics pathways underlying T cell activation. Immunity.

[bib217] Tanay A., Regev A. (2017). Scaling single-cell genomics from phenomenology to mechanism. Nature.

[bib218] Taniuchi I. (2018). CD4 helper and CD8 cytotoxic T cell differentiation. Annu. Rev. Immunol..

[bib219] Tegnér J., Björkegren J. (2007). Perturbations to uncover gene networks. Trends Genet..

[bib220] Thorsson V., Gibbs D.L., Brown S.D., Wolf D., Bortone D.S., Ou Yang T.H., Porta-Pardo E., Gao G.F., Plaisier C.L., Eddy J.A. (2018). The immune landscape of cancer. Immunity.

[bib221] Tikhonova A.N., Dolgalev I., Hu H., Sivaraj K.K., Hoxha E., Cuesta-Dominguez Á., Pinho S., Akhmetzyanova I., Gao J., Witkowski M. (2019). The bone marrow microenvironment at single-cell resolution. Nature.

[bib222] Tirosh I., Izar B., Prakadan S.M., Wadsworth M.H., Treacy D., Trombetta J.J., Rotem A., Rodman C., Lian C., Murphy G. (2016). Dissecting the multicellular ecosystem of metastatic melanoma by single-cell RNA-seq. Science.

[bib223] Topalian S.L., Drake C.G., Pardoll D.M. (2015). Immune checkpoint blockade: a common denominator approach to cancer therapy. Cancer Cell.

[bib224] Trapnell C., Cacchiarelli D., Grimsby J., Pokharel P., Li S., Morse M., Lennon N.J., Livak K.J., Mikkelsen T.S., Rinn J.L. (2014). The dynamics and regulators of cell fate decisions are revealed by pseudotemporal ordering of single cells. Nat. Biotechnol..

[bib270] Tusi B.K., Wolock S.L., Weinreb C., Hwang Y., Hidalgo L., Zilionis R., Waisman A., Huh J.R., Klein A.M., Socolovsky M. (2018). Population snapshots predict early haematopoietic and erythroid hierarchies. Nature.

[bib225] Velten L., Haas S.F., Raffel S., Blaszkiewicz S., Islam S., Hennig B.P., Hirche C., Lutz C., Buss E.C., Nowak D. (2017). Human haematopoietic stem cell lineage commitment is a continuous process. Nat. Cell Biol..

[bib226] Vento-Tormo R., Efremova M., Botting R.A., Turco M.Y., Vento-Tormo M., Meyer K.B., Park J.E., Stephenson E., Polański K., Goncalves A. (2018). Single-cell reconstruction of the early maternal–fetal interface in humans. Nature.

[bib227] Vierstra J., Stamatoyannopoulos J.A. (2016). Genomic footprinting. Nat. Methods.

[bib228] Vinayagam A., Stelzl U., Foulle R., Plassmann S., Zenkner M., Timm J., Assmus H.E., Andrade-Navarro M.A., Wanker E.E. (2011). A directed protein interaction network for investigating intracellular signal transduction. Sci. Signal..

[bib229] Voisinne G., Kersse K., Chaoui K., Lu L., Chaix J., Zhang L., Goncalves Menoita M., Girard L., Ounoughene Y., Wang H. (2019). Quantitative interactomics in primary T cells unveils TCR signal diversification extent and dynamics. Nat. Immunol..

[bib230] Wang B., Zhu J., Pierson E., Ramazzotti D., Batzoglou S. (2017). Visualization and analysis of single-cell RNA-seq data by kernel-based similarity learning. Nat. Methods.

[bib231] Wang Y.B., You Z.H., Li X., Jiang T.H., Chen X., Zhou X., Wang L. (2017). Predicting protein–protein interactions from protein sequences by a stacked sparse autoencoder deep neural network. Mol. Biosyst..

[bib232] Wei J., Long L., Zheng W., Dhungana Y., Lim S.A., Guy C., Wang Y., Wang Y.D., Qian C., Xu B. (2019). Targeting REGNASE-1 programs long-lived effector T cells for cancer therapy. Nature.

[bib233] Willis S.N., Nutt S.L. (2019). New players in the gene regulatory network controlling late B cell differentiation. Curr. Opin. Immunol..

[bib234] Wong M.T., Ong D.E., Lim F.S., Teng K.W., McGovern N., Narayanan S., Ho W.Q., Cerny D., Tan H.K., Anicete R. (2016). A high-dimensional atlas of human T cell diversity reveals tissue-specific trafficking and cytokine signatures. Immunity.

[bib235] Xia C., Fan J., Emanuel G., Hao J., Zhuang X. (2019). Spatial transcriptome profiling by MERFISH reveals subcellular RNA compartmentalization and cell cycle-dependent gene expression. Proc. Natl. Acad. Sci. U S A.

[bib236] Yamamoto R., Morita Y., Ooehara J., Hamanaka S., Onodera M., Rudolph K.L., Ema H., Nakauchi H. (2013). Clonal analysis unveils self-renewing lineage-restricted progenitors generated directly from hematopoietic stem cells. Cell.

[bib237] Yang K., Han X. (2016). Lipidomics: techniques, applications, and outcomes related to biomedical sciences. Trends Biochem. Sci..

[bib266] Yan H., Hale J., Jaffray J., Li J., Wang Y., Huang Y., An X., Hillyer C., Wang N., Kinet S. (2018). Developmental differences between neonatal and adult human erythropoiesis. Am. J. Hematol..

[bib238] Yang Z., Algesheimer R., Tessone C.J. (2016). A comparative analysis of community detection algorithms on artificial networks. Sci. Rep..

[bib239] Yona S., Kim K.W., Wolf Y., Mildner A., Varol D., Breker M., Strauss-Ayali D., Viukov S., Guilliams M., Misharin A. (2013). Fate mapping reveals origins and dynamics of monocytes and tissue macrophages under homeostasis. Immunity.

[bib240] Yoshida H., Lareau C.A., Ramirez R.N., Rose S.A., Maier B., Wroblewska A., Desland F., Chudnovskiy A., Mortha A., Dominguez C. (2019). The cis-regulatory atlas of the mouse immune system. Cell.

[bib262] You Y., Cuevas-Diaz Duran R., Jiang L., Dong X., Zong S., Snyder M., Wu J.Q. (2018). An integrated global regulatory network of hematopoietic precursor cell self-renewal and differentiation. Integr Biol. (Camb.).

[bib241] Yu H., Greenbaum D., Xin Lu H., Zhu X., Gerstein M. (2004). Genomic analysis of essentiality within protein networks. Trends Genet..

[bib242] Yu H., Kim P.M., Sprecher E., Trifonov V., Gerstein M. (2007). The importance of bottlenecks in protein networks: correlation with gene essentiality and expression dynamics. PLoS Comput. Biol..

[bib243] Yuan D., Tao Y., Chen G., Shi T. (2019). Systematic expression analysis of ligand-receptor pairs reveals important cell-to-cell interactions inside glioma. Cell Commun. Signal..

[bib244] Zeng H., Yu M., Tan H., Li Y., Su W., Shi H., Dhungana Y., Guy C., Neale G., Cloer C. (2018). Discrete roles and bifurcation of PTEN signaling and mTORC1-mediated anabolic metabolism underlie IL-7–driven B lymphopoiesis. Sci. Adv..

[bib245] Zhang B., Wang J., Wang X., Zhu J., Liu Q., Shi Z., Chambers M.C., Zimmerman L.J., Shaddox K.F., Kim S. (2014). Proteogenomic characterization of human colon and rectal cancer. Nature.

[bib246] Zhang P., Yang M., Zhang Y., Xiao S., Lai X., Tan A., Du S., Li S. (2019). Dissecting the single-cell transcriptome network underlying gastric premalignant lesions and early gastric cancer. Cell Rep.

[bib247] Zhang Q., He Y., Luo N., Patel S.J., Han Y., Gao R., Modak M., Carotta S., Haslinger C., Kind D. (2019). Landscape and dynamics of single immune cells in hepatocellular carcinoma. Cell.

[bib248] Zhang W., Chen Y., Li D., Yue X. (2018). Manifold regularized matrix factorization for drug-drug interaction prediction. J. Biomed. Inform..

[bib249] Zhou J.X., Taramelli R., Pedrini E., Knijnenburg T., Huang S. (2017). Extracting intercellular signaling network of cancer tissues using ligand-receptor expression patterns from whole-tumor and single-cell transcriptomes. Sci. Rep..

[bib250] Zhu C., Preissl S., Ren B. (2020). Single-cell multimodal omics: the power of many. Nat. Methods.

[bib251] Zhu X., Li H.D., Xu Y., Guo L., Wu F.X., Duan G., Wang J. (2019). A hybrid clustering algorithm for identifying cell types from single-cell RNA-seq data. Genes (Basel).

[bib252] Zhu Y., Clair G., Chrisler W.B., Shen Y., Zhao R., Shukla A.K., Moore R.J., Misra R.S., Pryhuber G.S., Smith R.D. (2018). Proteomic analysis of single mammalian cells enabled by microfluidic nanodroplet sample preparation and ultrasensitive nanoLC-MS. Angew. Chem. Int. Ed..

